# Long-term outcomes for neoadjuvant versus adjuvant chemotherapy in early breast cancer: meta-analysis of individual patient data from ten randomised trials

**DOI:** 10.1016/S1470-2045(17)30777-5

**Published:** 2018-01

**Authors:** Early Breast Cancer Trialists' Collaborative Group (EBCTCG), Bernard Asselain, William Barlow, John Bartlett, Jonas Bergh, Elizabeth Bergsten-Nordström, Judith Bliss, Francesco Boccardo, Clare Boddington, Jan Bogaerts, Gianni Bonadonna, Rosie Bradley, Etienne Brain, Jeremy Braybrooke, Philippe Broet, John Bryant, Julie Burrett, David Cameron, Mike Clarke, Alan Coates, Robert Coleman, Raoul Charles Coombes, Candace Correa, Joe Costantino, Jack Cuzick, David Danforth, Nancy Davidson, Christina Davies, Lucy Davies, Angelo Di Leo, David Dodwell, Mitch Dowsett, Fran Duane, Vaughan Evans, Marianne Ewertz, Bernard Fisher, John Forbes, Leslie Ford, Jean-Claude Gazet, Richard Gelber, Lucy Gettins, Luca Gianni, Michael Gnant, Jon Godwin, Aron Goldhirsch, Pamela Goodwin, Richard Gray, Daniel Hayes, Catherine Hill, James Ingle, Reshma Jagsi, Raimund Jakesz, Sam James, Wolfgang Janni, Hui Liu, Zulian Liu, Caroline Lohrisch, Sibylle Loibl, Liz MacKinnon, Andreas Makris, Eleftherios Mamounas, Gurdeep Mannu, Miguel Martín, Simone Mathoulin, Louis Mauriac, Paul McGale, Theresa McHugh, Philip Morris, Hirofumi Mukai, Larry Norton, Yasuo Ohashi, Ivo Olivotto, Soon Paik, Hongchao Pan, Richard Peto, Martine Piccart, Lori Pierce, Philip Poortmans, Trevor Powles, Kathy Pritchard, Joseph Ragaz, Vinod Raina, Peter Ravdin, Simon Read, Meredith Regan, John Robertson, Emiel Rutgers, Suzy Scholl, Dennis Slamon, Lidija Sölkner, Joseph Sparano, Seth Steinberg, Rosemary Sutcliffe, Sandra Swain, Carolyn Taylor, Andrew Tutt, Pinuccia Valagussa, Cornelis van de Velde, Jos van der Hage, Giuseppe Viale, Gunter von Minckwitz, Yaochen Wang, Zhe Wang, Xiang Wang, Tim Whelan, Nicholas Wilcken, Eric Winer, Norman Wolmark, William Wood, Milvia Zambetti, Jo Anne Zujewski

## Abstract

**Background:**

Neoadjuvant chemotherapy (NACT) for early breast cancer can make breast-conserving surgery more feasible and might be more likely to eradicate micrometastatic disease than might the same chemotherapy given after surgery. We investigated the long-term benefits and risks of NACT and the influence of tumour characteristics on outcome with a collaborative meta-analysis of individual patient data from relevant randomised trials.

**Methods:**

We obtained information about prerandomisation tumour characteristics, clinical tumour response, surgery, recurrence, and mortality for 4756 women in ten randomised trials in early breast cancer that began before 2005 and compared NACT with the same chemotherapy given postoperatively. Primary outcomes were tumour response, extent of local therapy, local and distant recurrence, breast cancer death, and overall mortality. Analyses by intention-to-treat used standard regression (for response and frequency of breast-conserving therapy) and log-rank methods (for recurrence and mortality).

**Findings:**

Patients entered the trials from 1983 to 2002 and median follow-up was 9 years (IQR 5–14), with the last follow-up in 2013. Most chemotherapy was anthracycline based (3838 [81%] of 4756 women). More than two thirds (1349 [69%] of 1947) of women allocated NACT had a complete or partial clinical response. Patients allocated NACT had an increased frequency of breast-conserving therapy (1504 [65%] of 2320 treated with NACT *vs* 1135 [49%] of 2318 treated with adjuvant chemotherapy). NACT was associated with more frequent local recurrence than was adjuvant chemotherapy: the 15 year local recurrence was 21·4% for NACT versus 15·9% for adjuvant chemotherapy (5·5% increase [95% CI 2·4–8·6]; rate ratio 1·37 [95% CI 1·17–1·61]; p=0·0001). No significant difference between NACT and adjuvant chemotherapy was noted for distant recurrence (15 year risk 38·2% for NACT *vs* 38·0% for adjuvant chemotherapy; rate ratio 1·02 [95% CI 0·92–1·14]; p=0·66), breast cancer mortality (34·4% *vs* 33·7%; 1·06 [0·95–1·18]; p=0·31), or death from any cause (40·9% *vs* 41·2%; 1·04 [0·94–1·15]; p=0·45).

**Interpretation:**

Tumours downsized by NACT might have higher local recurrence after breast-conserving therapy than might tumours of the same dimensions in women who have not received NACT. Strategies to mitigate the increased local recurrence after breast-conserving therapy in tumours downsized by NACT should be considered—eg, careful tumour localisation, detailed pathological assessment, and appropriate radiotherapy.

**Funding:**

Cancer Research UK, British Heart Foundation, UK Medical Research Council, and UK Department of Health.

## Introduction

Neoadjuvant chemotherapy (NACT)—ie, chemotherapy begun before breast cancer surgery—was introduced in the 1970s,^1^ aiming to downstage locally advanced (inoperable) disease and make it operable. NACT was subsequently extended to operable (early) breast cancer, mainly to allow breast-conserving surgery, and is now widely used, particularly for large tumours.^2^, ^3^, ^4^ Furthermore, NACT might be somewhat more likely to eradicate micrometastatic disease than might chemotherapy delayed until after surgery.

NACT might mitigate the hypothesised stimulatory effect of surgery on occult disease^5^ and reduce tumour cell shedding during surgery. NACT might also provide useful in-vivo information about the chemosensitivity of the local (and, by implication, disseminated) tumour to different chemotherapy regimens, helping to guide subsequent drug selection.^6^, ^7^ Conversely, by delaying surgery, NACT might increase the risk of metastatic spread, particularly for chemoresistant tumours.

Several randomised trials^8^, ^9^, ^10^, ^11^, ^12^, ^13^, ^14^, ^15^, ^16^, ^17^ have compared NACT with the same chemotherapy given postoperatively. Interpretation of these trials is complicated, however, as the frequency of breast-conserving surgery often differed between groups because of tumour shrinkage after NACT. In certain trials,^14^, ^15^ some good responders to NACT did not receive surgery, and high frequencies of local recurrence with NACT in these trials have been attributed to omission of definitive local therapy. Any such differences in the extent of surgery confound comparisons of the efficacy of NACT with that of adjuvant chemotherapy.^18^, ^19^ Another complication is that investigations of the influence of tumour characteristics on outcome need to use prerandomisation data, as analyses by postsurgical characteristics would be substantially biased by downstaging.^20^ To investigate such issues in more detail than was possible in reviews^18^, ^19^ of published data, we did a patient-level meta-analysis of the trials that directly compared any NACT regimen with the same regimen begun postoperatively.

**Research in context**

**Evidence before this study**

The Early Breast Cancer Trialists' Collaborative Group's ongoing extensive searches of bibliographic databases, including MEDLINE, Embase, the Cochrane Library, and meeting abstracts up to March 2017, identified 16 trials that compared neoadjuvant chemotherapy (NACT) with the same chemotherapy postoperatively. Meta-analyses of published reports indicate that NACT reduced the frequency of mastectomy but did not affect mortality. Interpretation is complicated, however, as the use of breast-conserving surgery often differed between groups because of tumour shrinkage by NACT. In certain trials, some patients with a good response did not receive surgery. Hence, women allocated NACT retained more breast tissue than did those allocated adjuvant chemotherapy, and higher local recurrence frequencies in some neoadjuvant trials than in others have been attributed to omission of definitive local therapy.

**Added value of this study**

We did a meta-analysis of individual patient data from trials that compared NACT with the same chemotherapy given postoperatively. We assessed effects of patient and tumour characteristics on tumour response, extent of local therapy, local and distant recurrence, breast cancer death, and overall mortality. This individual patient data meta-analysis, involving 4756 women in ten trials, found that the frequencies of clinical response and breast-conserving therapy were higher for smaller, higher-grade, and oestrogen receptor-negative and progesterone receptor-negative tumours, and for one trial using anthracycline and taxane chemotherapy. Although responders to NACT had lower distant recurrence and breast cancer mortality than did non-responders, when responders and non-responders were combined, distant recurrence and breast cancer mortality were similar for NACT and adjuvant chemotherapy. Local recurrence was, however, higher with NACT than with adjuvant chemotherapy, which persisted for 10 years after treatment and was not confined to trials in which surgery could be omitted after response to NACT.

**Implications of all the available evidence**

NACT is as effective as adjuvant chemotherapy in reducing the risk of distant recurrence and death from breast cancer. However, NACT is associated with higher local recurrence than adjuvant chemotherapy, which could be at least partly explained by wider use of breast-conserving therapy after NACT than with postoperative chemotherapy. Strategies to mitigate the increased local recurrence after breast-conserving therapy in tumours downsized by NACT should be considered—eg, careful tumour localisation, detailed pathological assessment, and appropriate radiotherapy.

## Methods

### Study design and participants

We sought data from all randomised trials in early (ie, operable) breast cancer that began before 2005 and compared NACT with the same chemotherapy begun after surgery (ie, standard adjuvant chemotherapy). NACT always started before surgery, although in some trials, some of the chemotherapy in the NACT group was given postoperatively, whereas all chemotherapy in the control group had to be postoperative (so trials such as NSABP B-27^21^ were ineligible). Trial identification and data checking were as reported previously^22^, ^23^ and conformed to the Preferred Reporting Items for Systematic Review and Meta-Analyses (Individual Patient Data).^24^ For every woman, we requested information from the trial's principal investigator or another appropriate member of their research group about patient and tumour characteristics, treatments, dates of any local recurrence (breast, chest wall, or regional nodes), distant recurrence, contralateral breast or other second primary cancer, and date last known to be alive or date and underlying cause of death. To avoid bias, we sought data for tumour characteristics recorded before randomisation since these characteristics can be altered by neoadjuvant treatment. We also requested tumour response after NACT (assessed mostly by palpation and mammography). To investigate the influence of NACT on extent of surgery, we sought details of surgery planned at randomisation and surgery actually done. When planned surgery was unknown, it was inferred from clinical tumour size. Patient-level data for radiotherapy were unavailable.

### Statistical analysis

A detailed description of the statistical methods has been previously published.^22^ Primary outcomes assessed were tumour response (complete response [no clinical evidence of disease after NACT], partial response [≥50% reduction in initial size], or stable or progressive disease [<50% reduction, no change, or increased tumour size]), extent of local therapy (mastectomy, lumpectomy [either with or without radiotherapy], and radiotherapy alone), local and distant recurrence, breast cancer death (via subtraction of the log-rank statistics of death without recurrence from those of overall survival^23^), and overall mortality (death from any cause). Analyses were by intention to treat and are of first isolated local recurrence (site not generally available), any distant recurrence (irrespective of previous local or contralateral recurrence), breast cancer mortality, and all-cause mortality. We treated deaths from new cancers of unknown primary sites as breast cancer deaths. When no recurrence was reported before breast cancer death, we assumed distant recurrence to have just preceded it. We took deaths from an unknown cause without recorded recurrence to be non-breast cancer deaths. Comparisons of NACT response and frequency of breast-conserving therapy used regression models,^25^ accounting for tumour size and trial. We stratified log-rank analyses by trial, follow-up year, age at entry (<35 years, 35–44 years, 45–54 years, 55–69 years, and ≥70 years), and prerandomisation clinical nodal status (N0 or other).

In such analyses, if a log-rank statistic (o–e) has variance v, then, defining z=(o–e)/√v and b=(o–e)/v, the event rate ratio (RR; NACT *vs* control) is estimated as exp(b) with SE=(RR–1)/z. RRs and confidence limits for RR are derived from those for b (by normal approximations). To test for a trend between n strata (eg, of age) in the effects of treatment, we supposed that stratum number s (s=1,2,…,n) has log-rank statistics (o–e) and v (with grand total over all strata O–E and V). We defined m, the mean stratum number, to be the sum, one term per stratum, of sv/V and define T to be the sum, one term per stratum, of (s–m)(o–e).^26^ The variance of T, var(T), is then the sum, one term per stratum, of (s–m)^2^/v. The trend test statistic (ie, the change from one stratum to the next in the log of the event RR) is then T/var(T), which has variance 1/var(T). Tests of whether two trends are the same involve subtraction of the corresponding trend test statistics from each other. A χ^2^ statistic on one degree of freedom (χ^2^_1_) for testing of whether some quantity Q differs significantly from zero is given by Q^2^/var(Q). A χ^2^ test (on n–1 degrees of freedom) for heterogeneity can be obtained by subtracting (O–E)^2^/V from the sum of the separate values, one per stratum, of (o–e)^2^/v. For analyses by regression, we estimated RRs by maximum likelihood; tests for trend and heterogeneity were by likelihood ratio.

Associations between baseline variables and outcome used prerandomisation values. Only two trials provided pathological response data, so correlations of characteristics with response to NACT use clinical response data (available for eight of ten trials). Subgroup analyses compare outcomes in trials in which all women allocated NACT were, or were not, scheduled to receive breast surgery and in women whose initially planned local treatment was mastectomy or breast-conserving therapy (lumpectomy with or without radiotherapy or radiotherapy alone). Sensitivity analyses assess the potential effect^27^ on local recurrence of competing events (distant recurrence and death without recurrence) and of omission of trials with only first recurrences recorded. p values of 0·05 or less are described as significant. Analyses used Stata 13.1 and R 2.13.2.

### Data sharing

Procedures for data access are available online.

### Role of the funding source

The funders of the study had no role in study design, data analysis, data interpretation, or writing of the report. The secretariat had full access to all the data in the study. The writing committee had final responsibility for the decision to submit for publication.

## Results

Individual patient data were available from ten^8^, ^9^, ^10^, ^11^, ^12^, ^13^, ^14^, ^15^, ^16^, ^17^ of 16 eligible trials identified and from 4756 (91%) of the 5250 women in total (table 1, appendix pp 2, 6, 17). Trial entry year for participants was 1983–2002, median follow-up was 9 years (IQR 5–14), with the last follow-up in 2013, and median age was 49 years (43–57). 1604 deaths occurred, including 248 (15%) without recurrence. Of the 4756 women included in the analysis, 3838 (81%) were in trials of regimens that included an anthracycline, one of which (902 women) also gave a taxane.^13^ Four trials (918 women) used MMM (mitoxantrone, methotrexate, and mitomycin-C)^11^, ^12^ or CMF (cyclophosphamide, methotrexate, and fluorouracil)^8^, ^17^ as NACT; in these trials, some chemotherapy in those allocated NACT was given after surgery (table 1, appendix pp 3–4). No patients received trastuzumab.

**Table 1:** Trials of neoadjuvant versus adjuvant chemotherapy that began by 2005

	Trials (n)*	**Women (n)**	**Deaths (n)**†	**Median years per woman (IQR)**	**Woman-years by years since entry (thousands)**			
					<10	10–19	≥20	Total
No anthracycline or taxane^8^, ^11^, ^12^, ^17^‡	4	918	315	7·0 (4·2–9·3)	6·0	0·8	0·2	7·0
Anthracycline, no taxane^9^, ^10^, ^14^, ^15^, ^16^	5	2936	1163	10·2 (4·9–15·4)	22·1	7·7	<0·1	29·8
Anthracycline and taxane^13^	1	902	126	7·9 (5·0–10·7)	6·5	0·5	0	7·0
Total	10	4756	1604	8·6 (4·8–13·7)	34·6	9·0	0·2	43·7

* Data are missing for six small trials that randomised about 500 women, so they were not included in this analysis (appendix p 17).

† Includes 1356 deaths with recurrence, 72 of unknown cause without recurrence, and 176 of known cause without recurrence.

‡ In these trials, women allocated to the neoadjuvant group completed their chemotherapy after surgery.

**Figure 1:**
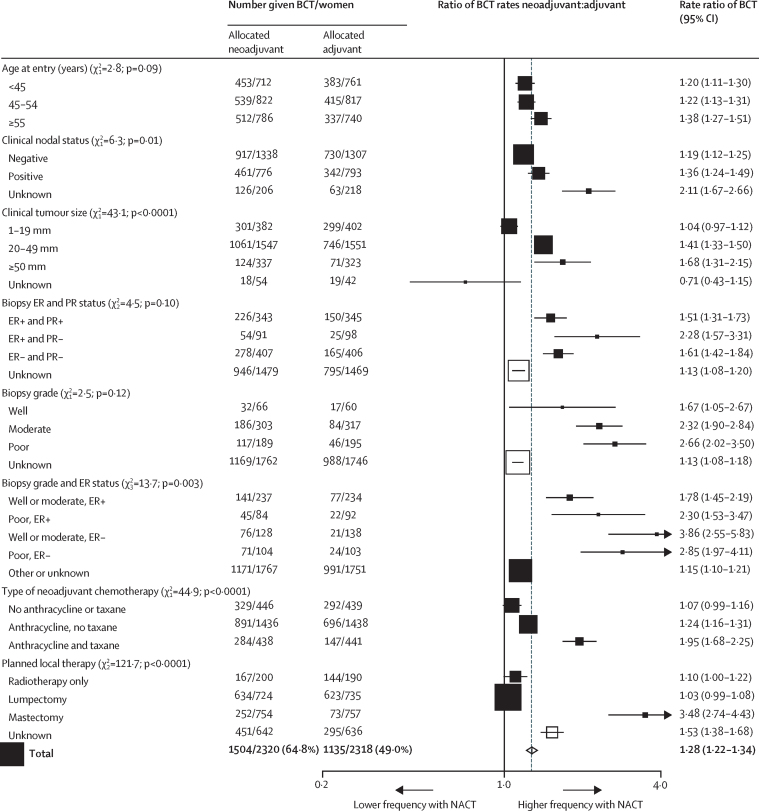
BCT rate ratios Numbers with BCT or mastectomy after chemotherapy. Excludes local therapy unknown (67 patients with NACT and 51 with adjuvant chemotherapies). BCT=breast-conserving therapy. ER=oestrogen receptor. PR=progesterone receptor. NACT=neoadjuvant chemotherapy.

**Figure 2:**
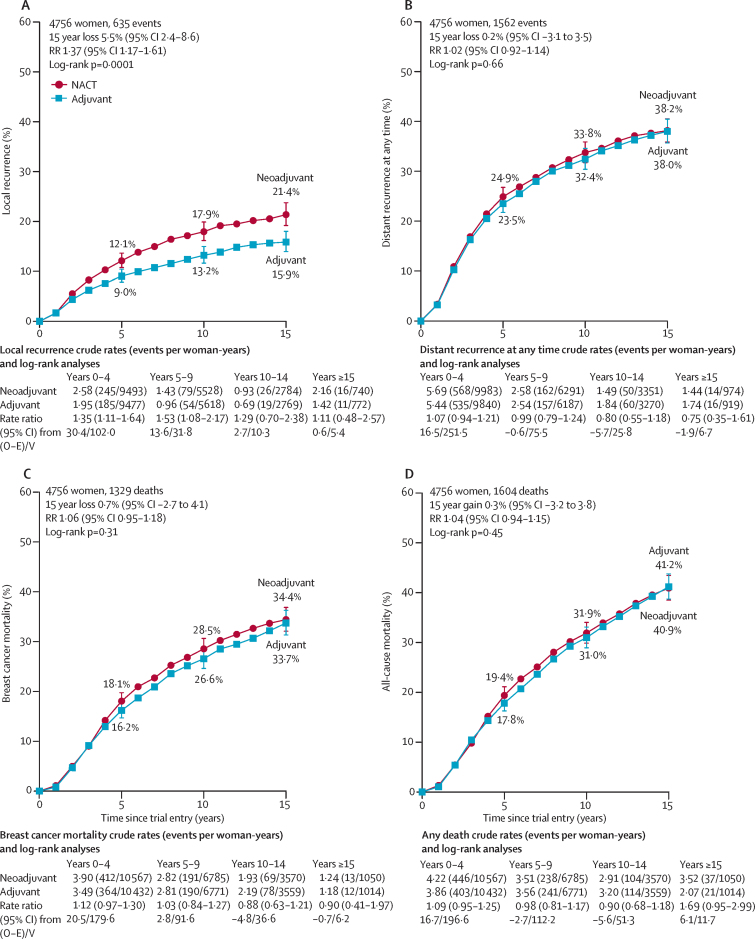
Effect of neoadjuvant versus adjuvant chemotherapy on recurrence and mortality Local recurrence (A), distant recurrence (B), breast cancer mortality (C), and death from any cause (D). Three trials recorded causes of any deaths but only the first breast cancer event. Hence, for these trials, distant recurrence includes the first distant recurrence as the first event and death from breast cancer. Error bars are 95% CIs. NACT=neoadjuvant chemotherapy. O–E=observed minus expected. RR=rate ratio. V=variance of O–E.

Across all trials, NACT was associated with substantial tumour response (table 2), moderately increased use of breast-conserving therapy in the NACT group compared with the adjuvant chemotherapy group (figure 1), and an absolute increase in 15 year local recurrence of 5·5% (95% CI 2·4–8·6; 21·4% for NACT *vs* 15·9% for adjuvant chemotherapy), corresponding to a RR of 1·37 (95% CI 1·17–1·61; p=0·0001; figure 2A). The incidence of local recurrence was significantly higher with NACT than with adjuvant chemotherapy in years 0–4 (RR 1·35 [95% CI 1·11–1·64]; p=0·003) and 5–9 (1·53 [1·08–2·17]; p=0·02), with few local recurrences after year 10. Sensitivity analyses indicated no substantial influence of competing risks from other breast events on the RRs for local recurrence (appendix p 18).

**Table 2:** Local therapy, planned versus done, in women allocated to neoadjuvant chemotherapy, by clinical response

**Therapy done**	**Clinical response**				
	Complete*	Partial†	Stable or progressive disease‡	Unknown	Total
**Planned breast-conserving therapy**					
Breast-conserving	215 (96%)	256 (90%)	119 (77%)	211 (81%)	801 (87%)
Mastectomy	10 (4%)	30 (10%)	35 (23%)	48 (19%)	123 (13%)
Unknown	0	0	0	2 (NA)	2 (NA)
Total response§	225/665 (34%)	286/665 (43%)	154/665 (23%)	261 (NA)	926 (100%)
**Planned mastectomy**					
Breast-conserving	75 (60%)	121 (41%)	30 (12%)	26 (36%)	252 (33%)
Mastectomy	49 (40%)	175 (59%)	231 (88%)	47 (64%)	502 (67%)
Unknown	0	1 (NA)	2 (NA)	11 (NA)	14 (NA)
Total response§	124/684 (18%)	297/684 (43%)	263/684 (38%)	84 (NA)	768 (100%)
**Unknown planned therapy**					
Breast-conserving	162 (83%)	164 (76%)	97 (56%)	28 (49%)	451 (70%)
Mastectomy	33 (17%)	53 (24%)	76 (44%)	29 (51%)	191 (30%)
Unknown	2 (NA)	3 (NA)	8 (NA)	38 (NA)	51 (NA)
Total response§	197/598 (33%)	220/598 (37%)	181/598 (30%)	95 (NA)	693 (100%)
**All women**					
Breast-conserving	452 (83%)	541 (68%)	246 (42%)	265 (68%)	1504 (65%)
Mastectomy	92 (17%)	258 (32%)	342 (58%)	124 (32%)	816 (35%)
Unknown	2 (NA)	4 (NA)	10 (NA)	51 (NA)	67 (NA)
Total response§	546/1947 (28%)	803/1947 (41%)	598/1947 (31%)	440 (NA)	2387 (100%)

Data are n (%) or n/N (%). NA=not applicable.

* No clinical evidence of disease.

† ≥50% reduction in tumour size.

‡ <50% reduction or increase in tumour size.

§ Percentages are of those with a known response.

**Figure 3:**
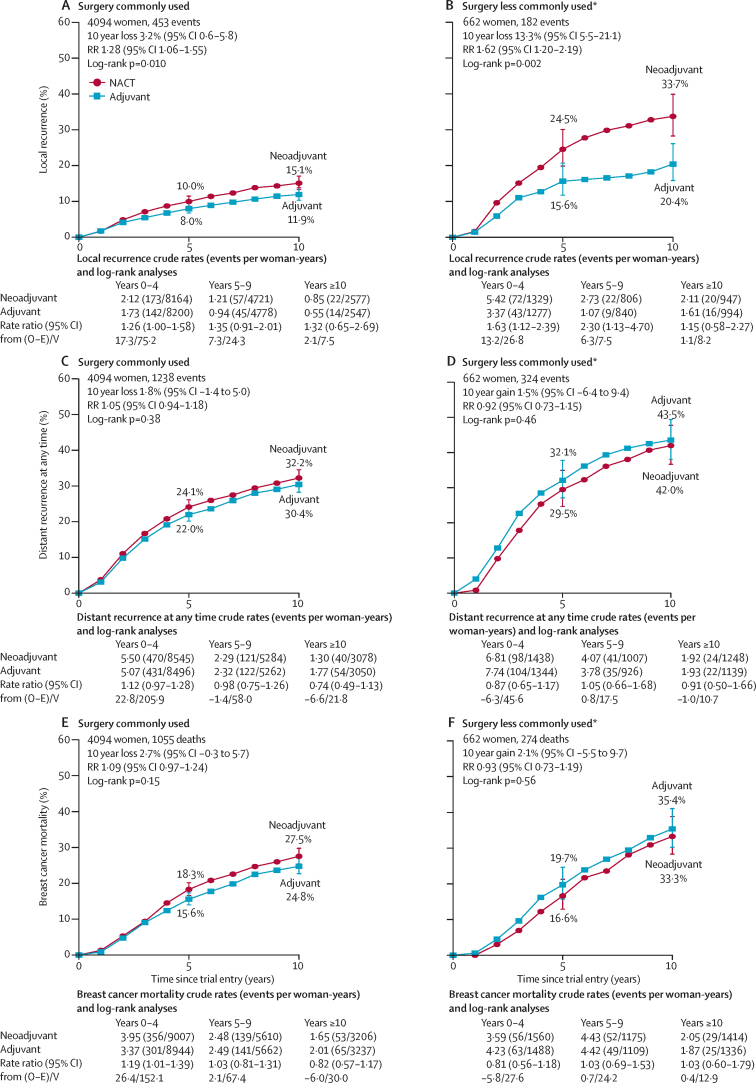
Time to recurrence and breast cancer mortality Local recurrence for surgery commonly used (A) and less commonly used (B), distant recurrence for surgery commonly used (C) and less commonly used (D), and breast cancer mortality for surgery commonly used (E) and less commonly used (F). Heterogeneity by surgery use: local recurrence p=0·19, distant recurrence p=0·29, and breast cancer mortality p=0·24. Error bars are 95% CIs. NACT=neoadjuvant chemotherapy. O–E=observed minus expected. RR=rate ratio. V=variance of O–E. Three trials recorded causes of any deaths but only the first breast cancer event. Hence, for these trials, distant recurrence includes the first distant recurrence as the first event and death from breast cancer. *Includes Institut Bergonié Bordeaux^14^ (in NACT group, 33% had radiotherapy alone) and Institut Curie S6^15^ (in NACT group, 51% had radiotherapy alone; in adjuvant chemotherapy group, 46% had radiotherapy alone) trials.

As anticipated,^18^, ^19^ the absolute increase in 10-year local recurrence with NACT was largest in the two trials^14^, ^15^ in which, after NACT, many women did not have breast surgery (13·3% [95% CI 5·5–21·1]; 33·7% for NACT *vs* 20·4% for adjuvant chemotherapy; RR 1·62 [95% CI 1·20–2·19], p=0·002; figure 3B). In the other eight trials,^8^, ^9^, ^10^, ^11^, ^12^, ^13^, ^16^, ^17^ surgery was scheduled irrespective of response to NACT, and the absolute increase in 10 year local recurrence was 3·2% (95% CI 0·6–5·8; 15·1% *vs* 11·9%; RR 1·28 [95% CI 1·06–1·55], p=0·01; figure 3A). However, the RRs for local recurrence in these two sets of trials were not significantly different (heterogeneity p=0·19).

**Figure 4:**
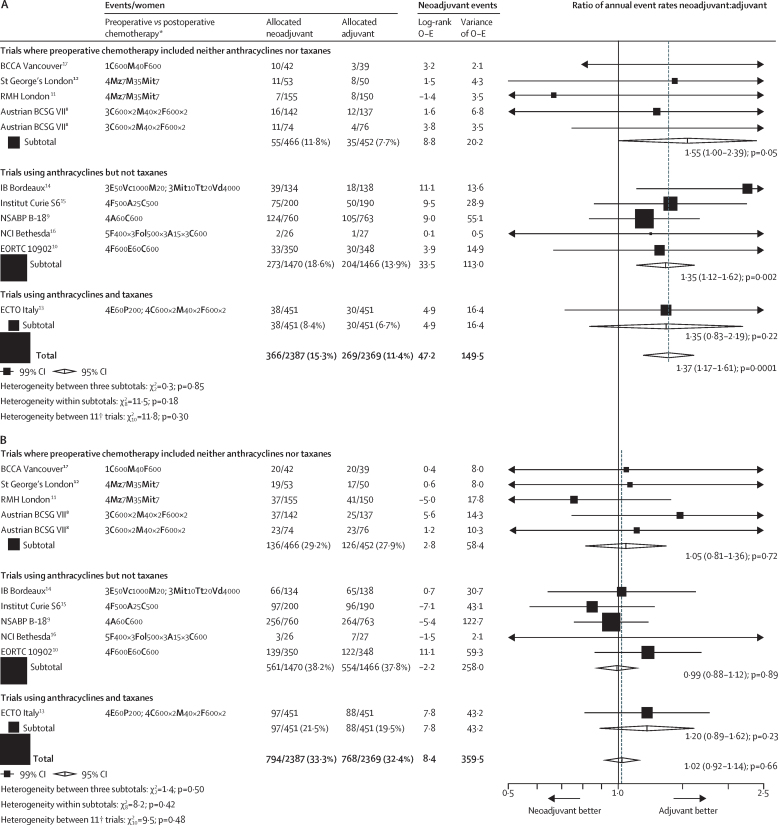
Rate ratios for the effect of neoadjuvant versus adjuvant chemotherapy on recurrence by trial (A) Local recurrence. (B) Distant recurrence. Three trials recorded causes of any deaths but only the first breast cancer event. Hence, for these trials, distant recurrence includes the first distant recurrence as the first event and death from breast cancer. The appendix (pp 3–4) contains a full description of each trial's chemotherapy regimen. A=doxorubicin (adriamycin). BCCA=British Columbia Cancer Agency. BCSG=Breast Cancer Study Group. C=cyclophosphamide. E=epirubicin. ECTO=European Cooperative Trial in Operable Breast Cancer. EORTC=European Organisation for Research and Treatment of Cancer. F=fluorouracil. Fol=folinic acid. IB=Institut Bergonié. M=methotrexate. Mit=mitomycin-C. Mz=mitoxantrone. NCI=National Cancer Institute. NSABP=National Surgical Adjuvant Breast and Bowel Project. O–E=observed minus expected. P=paclitaxel. Tt=thiotepa. Vc=vincristine. Vd=vindesine. *Chemotherapy regimens given preoperatively in those allocated neoadjuvant and postoperatively in those allocated adjuvant chemotherapy. The number of cycles, agents, and drug doses (in mg/m^2^) per cycle are given. †The Austrian BCSG VII trial8 has two entries to take into account the two postoperative chemotherapies given to both randomised groups (appendix pp 3–4).

**Figure 5:**
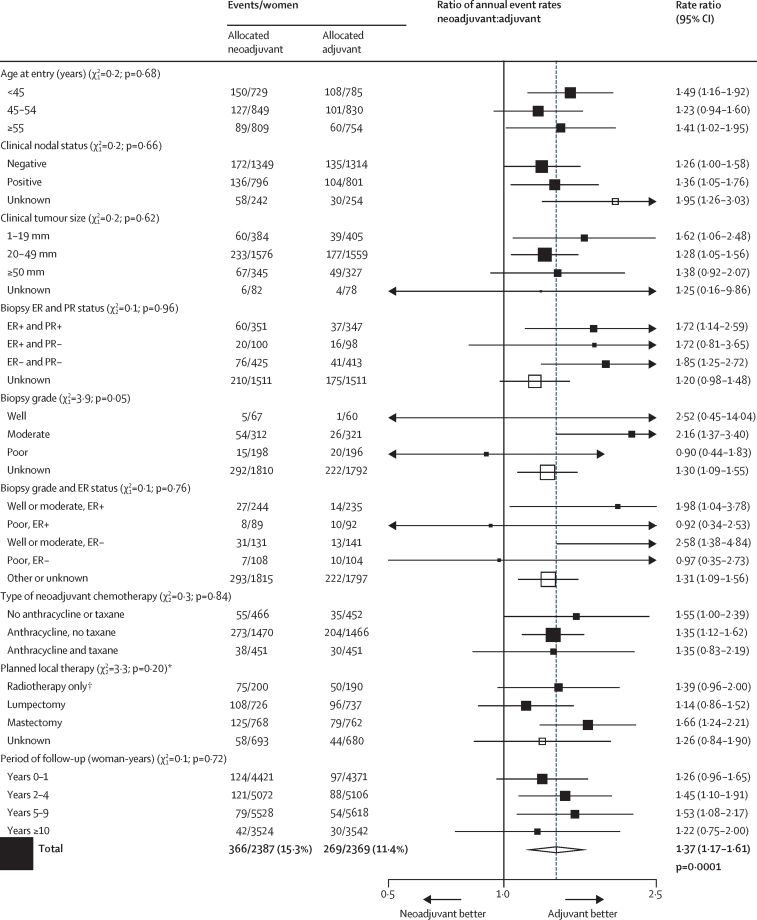
Local recurrence rate ratios For lumpectomy versus mastectomy, χ^2^_1_=3·3; p=0·07. ER=oestrogen receptor. PR=progesterone receptor. *408 women with missing data had planned local therapy imputed (appendix p 9). †Refers to Institut Curie S6^15^ (appendix p 9).

Between-trial RRs for local recurrence ranged from 0·67 (95% CI 0·24–1·91) to 4·59 (1·19–17·8), but this apparent heterogeneity was not significant (χ^2^_10_=11·8; p=0·30; figure 4A). RRs for local recurrence also did not differ significantly between the three classes of chemotherapy used in these trials (figure 4, appendix p 13), between trials in which chemotherapy in the NACT group was or was not completed after local therapy (appendix p 13), or between use or not of tamoxifen (figure 5, appendix p 13).

We noted no significant differences between NACT and adjuvant treatment in 15 year distant recurrence (38·2% for NACT *vs* 38·0% for adjuvant chemotherapy; RR 1·02 [95% CI 0·92–1·14]; p=0·66), breast cancer death (34·4% *vs* 33·7%; 1·06 [0·95–1·18]; p=0·31), or death from any cause (40·9% *vs* 41·2%; 1·04 [0·94–1·15]; p=0·45; figure 2B, C, D). The RRs for these three outcomes did not differ significantly between any subgroups of trials, including those for which use of surgery was or was not dependent on response to NACT, those using different types of chemotherapy, or those using or not using tamoxifen (figure 3 C–F and figure 4B, appendix pp 7, 13). Three trials^8^, ^9^, ^16^ collected only first recurrence and death rather than all events; however, sensitivity analyses omitting these trials had no material effect on distant recurrence estimates (appendix p 18). Mortality from causes other than breast cancer was no different between the NACT and adjuvant chemotherapy groups (appendix p 7).

Information about clinical tumour response was available for 1947 (82%) of 2387 patients allocated NACT; 546 (28%) of 1947 had a complete response, 803 (41%) of 1947 had a partial response, and 598 (31%) of 1947 had stable or progressive disease (table 2, appendix p 8). The clinical tumour response to NACT affected surgical treatment decisions: more women with a complete response had breast-conserving therapy (452 [83%] of 544) than did those with a partial response (541 [68%] of 799) or no response (246 [42%] of 588). Consequently, we noted an imbalance by treatment group in the extent of surgery: although breast-conserving therapy was initially intended for equal numbers of patients in each group, actual use of breast-preserving therapy (including no surgery) was 1504 (65%) of 2320 in the NACT group versus 1135 (49%) of 2318 in the adjuvant chemotherapy group excluding patients with unknown surgeries (p<0·0001; figure 1, appendix pp 9, 10).

**Figure 6:**
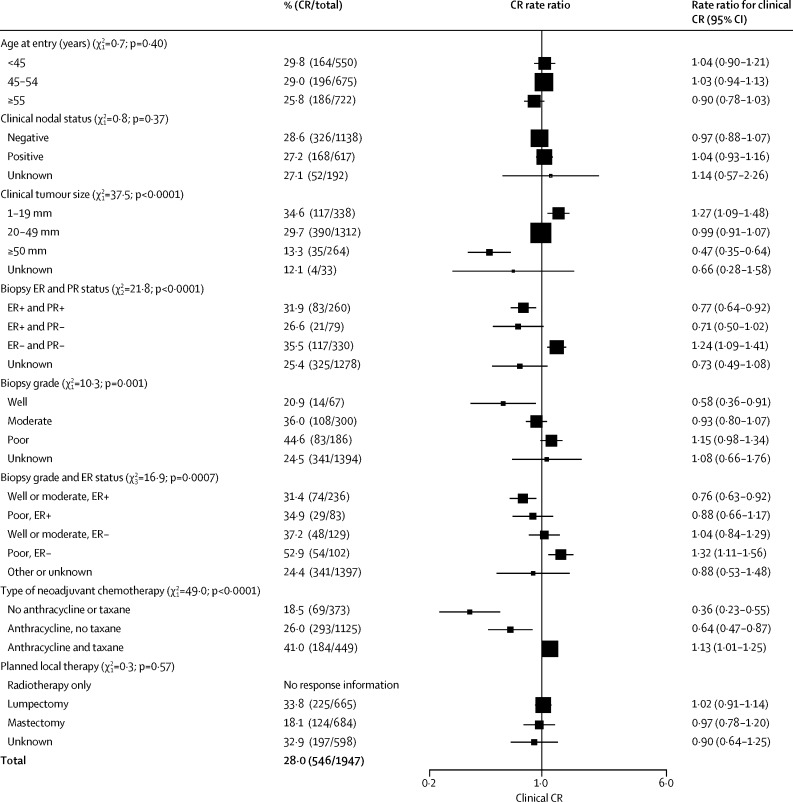
Clinical complete response rate ratios Three trials are excluded, as individual responses are not available; 440 women have missing clinical response data. CIs are group specific.^25^ Rate ratios are scaled such that, within each category, their inverse variance-weighted sum is 1—ie, ratios are with respect to the mean CR. The appendix (p 6) contains data available for each trial. CR=complete response. ER=oestrogen receptor. PR=progesterone receptor.

Figure 6 shows proportions of women with complete clinical response according to patient and tumour characteristics. Complete response decreased with increasing clinical tumour size (trend p<0·0001) and was higher with oestrogen receptor (ER)-negative biopsies than with ER-positive biopsies (p<0·0001) and with poorly differentiated tumours than with well or moderately differentiated tumours (trend p=0·001, even after allowance for high-grade tumours tending to be ER negative), and was higher in the one trial^13^ that combined anthracycline and taxane therapy than in the other trials (p<0·0001). Age, nodal status, and planned local therapy did not affect response.

The proportion of women having breast-conserving therapy in various different subgroups in the NACT and adjuvant chemotherapy groups are shown in figure 1. The strongest predictors of the effect of NACT on breast conservation frequency were tumour size, planned local therapy, and type of chemotherapy (all p<0·0001). The effect of NACT on surgery de-escalation was most apparent among women with large (20–49 mm or ≥50 mm) tumours; we noted little effect of NACT on breast conservation frequency in women with small (<20 mm) tumours. As expected, women with mastectomy originally planned were more likely to have lesser surgery than were those with breast-conserving surgery originally planned. NACT with an anthracycline and taxane combination was also associated with substantially more surgery de-escalation than was NACT with other regimens. In women with node-positive disease, the rate ratio for BCT was higher than for those with node-negative disease (p=0·01). Despite the high frequency of clinical response in patients with ER-negative and poorly differentiated tumours, ER status and tumour grade were not associated with frequency of breast-conserving therapy, although after accounting for grade, ER-negative women did appear to have higher breast-conserving frequencies. Age was not associated with the freqeuncy of breast-conserving therapy.

If the increased local recurrence in the NACT groups (figure 3) is due to de-escalation of local therapy from mastectomy to breast-conserving therapy, the RR for the effect on local recurrence of allocation to NACT should be greatest in women for whom mastectomy was originally planned. 252 (33%) of 754 women converted from planned mastectomy to breast-conserving therapy. However, although the RR for local recurrence among all women planned to have a mastectomy was 1·66 (95% CI 1·24–2·21) compared with 1·14 (0·86–1·52) for women with lumpectomy planned, the two-tailed test for heterogeneity was not significant (p=0·07; figure 5, appendix pp 11, 12).

Heterogeneity between the RRs for local recurrence was lower across all other tumour characteristics than it was for RRs in the subgroup by planned local therapy (figure 5); although the p value for the trend in RR with biopsy grade was 0·05, this p value could have been a chance finding given that it was the most extreme from many subgroup analyses. Despite surgery de-escalation being more common in larger tumours than in smaller tumours, and in the trial combining anthracycline and taxane^13^ than in trials of other regimens, the proportional increases in local recurrence did not vary significantly by tumour size or chemotherapy regimen (figure 5). RRs also did not differ by age, nodal status, ER or progesterone receptor status, or period of follow-up. Patient-level data for radiotherapy were not available, and trial-level data for radiotherapy intent and practice were incomplete (appendix p 2), so the effect of radiotherapy on local recurrence cannot be studied. Radiotherapy was scheduled for most women who had breast-conserving surgery and actual use of radiotherapy was more frequent in the NACT than in the adjuvant therapy groups (appendix p 2). The RRs for distant recurrence and breast cancer mortality did not vary by any tumour factor measured, type of chemotherapy, timing of chemotherapy use in the NACT group, type of planned local therapy, or period of follow-up (appendix p 13).

As expected, distant recurrence and breast cancer mortality were substantially lower in complete responders than in non-responders (appendix p 14). However, women who had a complete clinical response after NACT had a frequency of local recurrence similar to that of partial responders or non-responders. Ordering of trials by the percentage of patients with a complete clinical response to NACT did not reveal any significant trend of improved recurrence or breast cancer mortality RRs in trials with a higher frequency of response (appendix p 16). No patterns emerged between trials in complete response when considering the year that the trial started or the frequency of breast-conserving therapy within a trial (appendix p 15).

## Discussion

In early breast cancer, high frequencies of complete or partial clinical response can be achieved with NACT, which can lead to a higher frequency of breast-conserving therapy than with adjuvant chemotherapy. However, we found NACT to be associated with a higher frequency of local recurrence than was the same chemotherapy started after surgery. Reassuringly, the increase in local recurrence was not associated with any significant increase in distant recurrence or breast cancer mortality.

More than two thirds of patients receiving NACT responded, with more than a quarter achieving complete clinical response, despite some trials using old chemotherapy regimens and four administering some of the chemotherapy postoperatively. The one regimen that included both anthracycline and taxane had the highest frequency of complete response. Within trials, response was more common in women with small, ER-negative and progesterone receptor-negative, or high-grade tumours, as measured before randomisation, but was little affected by age, nodal status, or planned local therapy.^8^, ^9^, ^10^, ^11^, ^12^, ^13^, ^14^, ^15^, ^16^, ^17^ As expected, use of NACT was associated with an increase in the use of breast-conserving therapy.

An increase in the use of breast-conserving therapy in women who responded well to NACT and who would otherwise have had mastectomy is a likely explanation for the increase in local recurrence in patients allocated NACT. As anticipated,^18^, ^19^ the absolute increase in local recurrence was greatest in the two trials^14^, ^15^ in which surgery could be avoided completely in the event of a complete clinical response to NACT. This apparent heterogeneity of effect was, however, not significant, and NACT appeared to also have resulted in some increase in local recurrence in the aggregated results from the eight other trials. Hence, the increased local recurrence with NACT is not wholly explained by omission of surgery. Other unexamined factors might also have contributed to the increased local recurrence with NACT. For example, after NACT, tumour localisation can be difficult^28^ and response patterns can be heterogeneous,^29^ making surgery technically more difficult than without use of NACT. Differing use of radiotherapy or axillary surgery in the NACT group might also have contributed to the higher local recurrence, although patient-level information about this factor was not available. Trial reports indicate that radiotherapy was scheduled for most women who had breast-conserving surgery and that actual use of radiotherapy was, if anything, more frequent in the NACT than in the adjuvant therapy groups. Thus, lesser use of radiotherapy after NACT than that without NACT is unlikely to explain this increase in local recurrence. Indeed, even with radiotherapy, local failure is higher after breast-conserving surgery than after mastectomy without radiotherapy.^30^

Our finding of an overall increase in local recurrence in the trials using optimal local treatment is at odds with a meta-analysis^18^ based on published data rather than individual patient data, but this discrepancy could be because the meta-analysis included comparisons that were confounded by differing background systemic therapy.

Tumour response is predictive of lower distant recurrence and death than an absence of tumour response. Compared with all women randomly allocated to adjuvant chemotherapy, outcomes were better for those with a complete clinical response after NACT than for those with a partial response and far better than for those with little or no response to NACT. However, even in trials with high frequencies of complete response, NACT was not significantly better than adjuvant chemotherapy with respect to distant recurrence or breast cancer mortality. This finding could be because tumour characteristics that are associated with higher response—such as smaller tumour size—are also associated with lower distant recurrence and are balanced between the NACT and adjuvant groups by randomisation. In each trial, both groups eventually receive the same chemotherapy, so any differences between trials in the efficacy of chemotherapy regimens will apply to both groups. The CTNeoBC study^6^ reported similar findings in that efficacy as assessed by high pathological complete response at the trial level did not correlate well with long-term efficacy.

A limitation of our meta-analysis is that we have not been able to assess reliably whether presurgical systemic therapy is more effective at eradicating micrometastatic disease than the same chemotherapy administered after recovery from surgery because of the confounding effect of differences in the extent of surgery between women allocated NACT and those allocated adjuvant chemotherapy. A large trial with the same surgery and radiotherapy in the NACT and adjuvant chemotherapy groups could assess this question. At present, we cannot exclude the possibility that NACT does moderately reduce distant recurrence compared with the same chemotherapy given postoperatively, but that this benefit was obscured by an increase in local recurrence due to less extensive surgery after NACT than in patients who did not receive NACT. Trials of radiotherapy after surgery indicate that substantially decreasing local recurrence does also decrease breast cancer mortality, with about one breast cancer death prevented for every four local recurrences prevented.^30^ The small, non-significant excess of breast cancer mortality in patients allocated NACT is consistent with this risk ratio, so could therefore be due to the increase in local recurrence, but it could equally well be a chance finding.

The main aim of NACT in contemporary practice is to reduce the extent of breast surgery, thereby making breast conservation feasible in women who would otherwise need mastectomy. In the time since the trials in this meta-analysis were done, pathology reporting, surgery, and radiotherapy have improved, and more effective systemic neoadjuvant regimens have been introduced than were available when these trials took place. These changes should increase the likelihood of successful downstaging to allow conservative surgery in current and future practice. But, although improvements in treatment mean local recurrence risk should be lower than in these trials, our findings indicate that tumours downsized by NACT might continue to be associated with higher local recurrence risk after breast-conserving surgery than might tumours of the same dimensions in women who have not received NACT. Strategies to mitigate the increased local recurrence after breast-conserving therapy in tumours downsized by NACT should be considered—for example, careful tumour localisation, detailed pathological assessment, and appropriate radiotherapy.^31^ Prospective randomised trials would also help to establish the optimal clinical management in this context.

NACT allows more breast-conserving therapy than does adjuvant chemotherapy and provides information about an individual patient's response to a particular chemotherapy regimen. However, it appears to be no better than postoperative adjuvant treatment at reducing breast cancer mortality and, perhaps as a consequence of a reduction of the extent of surgery, NACT is associated with moderately increased local recurrence risk, which persists for at least 10 years.

Correspondence to: EBCTCG Secretariat, Medical Research Council Population Health Research Unit, Nuffield Department of Population Health, Oxford OX3 7LF, UK **bc.overview@ndph.ox.ac.uk**
