# Supplementary material for: Long-term outcomes for neoadjuvant versus adjuvant chemotherapy in early breast cancer: meta-analysis of individual patient data from ten randomised trials
Source: Lancet Oncol. 2018 Jan;19(1):27–39. doi: 10.1016/S1470-2045(17)30777-5 (PMC5757427; doi:10.1016/S1470-2045(17)30777-5)
Supplement: Supplementary appendix [file mmc1.pdf]

THE LANCET
Oncology

Supplementary appendix

This appendix formed part of the original submission and has been peer reviewed. We post it as supplied by the authors.

Supplement to: Early Breast Cancer Trialists' Collaborative Group (EBCTCG). Long-term outcomes for neoadjuvant versus adjuvant chemotherapy in early breast cancer: meta-analysis of individual patient data from ten randomised trials. *Lancet Oncol* 2017; published online Dec 11. http://dx.doi.org/10.1016/S1470-2045(17)30777-5.

Contents list of appendix to: Neo-adjuvant versus adjuvant chemotherapy in early breast cancer: patient-level meta-analysis of long-term outcomes among 4756 women in 10 randomised trials

CONTROL+CLICK on any page number to jump to it

		Page
**Materials**		
Webtable 1	Trial eligibility criteria, radiotherapy and surgery details.	2
Webtable 2	Trial systemic treatments and sequencing with surgery by allocated treatment	3
Webtable 3	Definitions of tumour response and tumour response assessment within trial	5
Webtable 4	Data items available by trial	6
**Mortality**		
Webfigure 1	Mortality by trial	7
**Tumour response**		
Webtable 5	Breast tumour clinical response	8
**Extent of local therapy**		
Webtable 6	Planned versus actual surgery by trial and allocated treatment	9
Webfigure 2	Planned versus actual surgery given by allocated treatment	10
**Recurrence and extent of local therapy**		
Webtable 7	Local recurrence in women randomised to neo-adjuvant chemotherapy by their sequence of planned and actual local therapy	11
Webfigure 3	Local recurrence by extent of planned local therapy and trial	12
**Recurrence and mortality by various factors**		
Webfigure 4	Effect of neo-adjuvant chemotherapy on recurrence and breast cancer mortality by various factors	13
**Recurrence and mortality by various factors**		
Webfigure 5	Recurrence and mortality by clinical response	14
**Tumour response, recurrence and mortality**		
Webfigure 6	Percentage of tumour complete clinical response by trial and breast-conserving surgery rates	15
Webfigure 7	Recurrence and breast cancer mortality by trial and by percentage of women with breast tumour complete clinical response and breast-conserving rate.	16
**Trials not available for analysis**		
Webtable 8	Details of trials not available for analysis.	17
**Sensitivity analyses**		
Webtable 9	Sensitivity analyses of using different methods to assess the affect of neo-adjuvant chemotherapy on local and distant recurrence.	18
**EBCTCG collaborators, alphabetically by institution and name**		
Webfigure 8	Collaborators	19

Webtable 1: Trial eligibility criteria, recruitment period, radiotherapy and surgery details

Trial, entry years	Eligibility	Radiotherapy (RT) sites: breast, chest wall, axilla, SCF, IMC	Type of surgery (RM, MRM, M, Lx=BCS, AD)
BCCA Vancouver (83-90)	Stages I and II, high risk only	Publication of pilot study indicates N+ women with inner or central quadrant tumours received RT, but no details of areas irradiated	Neoadjuvant – 66% had MRM, 31% Lx, 3% unknown<br>Adjuvant – 67% had MRM, 30% Lx, 3% unknown
IB Bordeaux (85-90)	Tumour T2-3, N0-1, M0	Neoadjuvant – B+nodes, IMC & SCF (CR), B (PR), and no RT if SD/PD (had M)<br>Adjuvant – no RT	Neoadjuvant – 37% MRM (SD/PD) , 30% Lx (PR), 33% None (CR)<br>Adjuvant – 100% MRM
Institut Curie S6 (86-90)	Tumour 3-7cm, N0-1, M0	Neoadjuvant – RT (pre-surgery) to B + boost if responded to RT<br>Adjuvant – RT (pre-surgery) to B + boost if responded to RT<br>All women 54 Gy to axilla + 10-15 Gy boost if N1 (if no surgery), 45 Gy to SCF/IMC	Neoadjuvant – 18% MRM±RT, 31% Lx+RT, 51% none<br>Adjuvant – 23% MRM±RT, 32% Lx+RT, 46% none<br>Surgery determined by tumour mass at time of surgery.
NSABP B-18 (88-93)	T1-3, N0-1	Neoadjuvant – RT to B after Lx, none after RM<br>Adjuvant – RT to B after Lx, none after RM	Neoadjuvant – 22% RM, 68% Lx+AD<br>Adjuvant – 40% RM, 60% Lx+AD<br>Lx likely if T ≤ 5cm and N0
St George's London (90-93)	Age 30-69, M0	T<5cm RT to B+boost and RT to axilla & SCF if N+. RT to IMC if medial tumour.<br>T>5cm, no surgery RT to B+optional boost to tumour site; RT to axilla & SCF if N+.	Neoadjuvant – 15% M, 66% Lx+AD1, 19% none<br>Adjuvant – 12% M, 84% Lx+AD1 , 4% none<br>M if T>5cm and no response to RT, Lx+RT if T<5cm, no surgery if T>5cm and response to RT
RMH London (90-95)	Age <70, operable	Neoadjuvant – RT to B +boost and RT to axilla & SCF if N+ after Lx, none after M<br>Adjuvant – RT to B + boost and RT to axilla & SCF if N+ after Lx, none after M	Neoadjuvant – 11% M, 88% Lx, 1% none<br>Adjuvant – 22% M, 77% Lx, 1% none<br>Both arms: surgery appropriate to the size and position of tumour and palpable axillary lymph nodes excised by lower axillary sampling.
NCI Bethesda (90-98)	T2N0-1 , T1N1	Neoadjuvant – RT to B+boost and RT to SCF in N+ after Lx, none after MRM<br>Adjuvant – RT to B+boost and RT to SCF if N+ after Lx, none after MRM	Neoadjuvant – 58% MRM, 42% Lx+AD<br>Adjuvant – 59% MRM, 41% Lx+AD
Austrian BCSG VII (91-99)	T1-3, N0-1, M0	Neoadjuvant – RT (targets unspecified) after Lx, RT discretionary after MRM<br>Adjuvant – RT (targets unspecified) after Lx, RT discretionary after MRM	Neoadjuvant – 34% MRM, 66% Lx+AD<br>Adjuvant – 40% MRM, 60% Lx+AD
EORTC 10902 (91-99)	T1c/2/3/4b, N0-1	Neoadjuvant – RT to B after Lx, RT to CW+IMC if T>5cm, +SCF if N+ after MRM<br>Adjuvant – RT to B after Lx, RT to CW+IMC if T>5cm, +SCF if N+ after MRM<br>RT in all cases if surgery not considered radical	Neoadjuvant – 58% MRM, 34% Lx+AD, 8% none<br>Adjuvant – 75% MRM, 23% Lx+AD , 2% none<br>Surgery determined by tumour mass at time of surgery.
ECTO Italy (96-02)	Operable breast cancer size >2cm	Neoadjuvant – RT to B+boost, RT after M if T4 disease<br>Adjuvant – RT to B+boost, RT after M if T4 disease.<br>Institutional variation accepted	Neoadjuvant – 33% MRM, 65% Lx+AD3, 2% none<br>Adjuvant – 66% MRM, 34% Lx+AD3, 0% none<br>Surgery determined by tumour mass at time of surgery.

ADn=axillary dissection (if given, n=level), B=breast CW=chest wall, CR=complete response, IMC=internal mammary chain, Lx=lumpectomy, M=mastectomy (details unspecified), MRM=modified radical mastectomy, PR=partial response, RM=radical mastectomy, RT=radiotherapy, SCF=supraclavicular fossa, SD/PD=stable/progressive disease.

Webtable 2: Trial systemic treatments and sequencing with surgery, by allocated treatment (N=Neo-adjuvant, A = Adjuvant only)

Trial	Arm	Treatment details
BCCA Vancouver	N	(Cyclophosphamide [600 mg/m^2 iv] + Methotrexate [40 mg/m^2 iv] + 5-Fluorouracil [600 mg/m^2 iv]) × 1 pre-op +<br>(Cyclophosphamide [600 mg/m^2 iv] + Methotrexate [40 mg/m^2 iv] + 5-Fluorouracil [600 mg/m^2 iv]) q3wk × 8 postop
	A	(Cyclophosphamide [600 mg/m^2 iv] + Methotrexate [40 mg/m^2 iv] + 5-Fluorouracil [600 mg/m^2 iv]) q3wk × 9 postop
IB Bordeaux	N	(Epirubicin [50 mg/m^2] + Vincristine [1 mg/m^2] + Methotrexate [20 mg/m^2]) q3wk × 3 pre-op +<br>(Mitomycin-C [10 mg/m^2] + Triethylenephosphoramide [20 mg/m2] + Vindesine [4 mg/m^2]) q3wk × 3 pre-op, then<br>Radiotherapy (in absence of residual tumour) or Radiotherapy + (Tumorectomy for < 2cm residual tumour or Patey Mastectomy for > 2cm residual tumour)
	A	(Epirubicin [50 mg/m^2] + Vincristine [1 mg/m^2] + Methotrexate [20 mg/m^2]) q3wk × 3 (if N+ or ER-poor or PR-poor) post-op +<br>(Mitomycin-C [10 mg/m^2] + Triethylenephosphoramide [20 mg/m^2] + Vindesine [4 mg/m^2]) q3wk × 3 for) postop
Institut Curie S6	N	(Cyclophosphamide [500 mg/m^2] + Doxorubicin [25 mg/m^2] + 5-Fluorouracil [500 mg/m^2]+ P) q4wk × 4 pre-op
	A	(Cyclophosphamide [500 mg/m^2] + Doxorubicin [25 mg/m^2] + 5-Fluorouracil [500 mg/m^2]+ P) q4wk × 4 postop
NSABP B-18	N	(Doxorubicin [60mg/m^2] + Cyclophosphamide [600mg/m^2]) q21d × 4 pre-op
	A	(Doxorubicin [60mg/m^2] + Cyclophosphamide [600mg/m^2]) q21d × 4 postop
NSABP B-18	N	(Doxorubicin [60mg/m^2] + Cyclophosphamide [600mg/m^2]) q21d × 4 pre-op + Tamoxifen [20mg/d] to year 5
	A	(Doxorubicin [60mg/m^2] + Cyclophosphamide [600mg/m^2]) q21d × 4 postop + Tamoxifen [20mg/d] to year 5
St George's London	N	(Mitoxantrone [7mg/m^2] + Methotrexate [30 mg/m^2]) q3wk × 4 + Mitomycin-C [7mg/m^2] q6wk × 2 + Folinic Acid [15mg × 4 in 24h, 24h after chemo] pre-op +<br>(Mitoxantrone [7mg/m^2] + Methotrexate [30 mg/m^2]) q3wk × 4 + Mitomycin-C [7mg/m^2] q6wk × 2 + Folinic Acid [15mg × 4 in 24h, 24h after chemo] postop if the patient responded to pre-op MMM otherwise (5-Fluorouracil [600mg/m^2] + Epirubicin [50mg/m^2] + Cyclophosphamide [600mg/m^2]) q3wk × 8 postop
	A	(Mitoxantrone [7mg/m^2] + Methotrexate [30 mg/m^2]) q3wk × 8 + Mitomycin-C [7mg/m^2] q6wk × 4 + Folinic Acid [15mg × 4 in 24h, 24h after chemo] postop
RMH London	N	(Mitoxantrone [7mg/m^2] + Methotrexate [35 mg/m^2]) q3wk × 4 + Mitomycin-C [7mg/m^2] q6wk × 2 + Tamoxifen [20 mg/d] pre-op +<br>(Mitoxantrone [7mg/m^2] + Methotrexate [35 mg/m^2]) q3wk × 4 + Mitomycin-C [7mg/m^2] q6wk × 2 postop + Tamoxifen [20 mg/d] to year 5
	A	(Mitoxantrone [7mg/m^2] + Methotrexate [35 mg/m^2]) q3wk × 8 + Mitomycin-C [7mg/m^2] q6wk × 4 postop + Tamoxifen [20 mg/d] to year 5
NCI Bethesda	N	(5-Fluorouracil [400 mg/m^2 iv] d1,2,3 + Folinic Acid [500 mg/m^2 iv d1,2,3 1 hour before 5-FU] + Doxorubicin [15 mg/m^2 iv] d1,2,3 +<br>Cyclophosphamide [600 mg/m2 iv] d1 + Mesna [200mg/m^2 iv] + {GM-CSF [10 µg/kg sc daily d4-16] or G-CSF [5 µg/kg sc daily d4-18]}) q3wk × 5 pre-op
	A	(5-Fluorouracil [400 mg/m^2 iv] d1,2,3 + Folinic Acid [500 mg/m^2 iv d1,2,3 1 hour before 5-FU] + Doxorubicin [15 mg/m^2 iv] d1,2,3 +<br>Cyclophosphamide [600 mg/m^2 iv] d1 + Mesna [200mg/m^2 iv] + {GM-CSF [10 µg/kg sc daily d4-16] or G-CSF [5 µg/kg sc daily d4-18]}) q3wk × 5 postop

Continued

Webtable 2 cont'd: Trial systemic treatments and sequencing with surgery, by allocated treatment (N=neo-adjuvant, A = Adjuvant only)

Trial	Arm	Treatment details
Austrian BCSG VII	N	(Cyclophosphamide [600mg/m² d1,8] + Methotrexate [40mg/m² d1,8] + 5-Fluorouracil [600mg/m² d1,8]) × 3 pre-op + (Cyclophosphamide [600mg/m² d1,8] + Methotrexate [40mg/m² d1,8] + 5-Fluorouracil [600mg/m² d1,8]) × 3 postop
	A	(Cyclophosphamide [600mg/m² d1,8] + Methotrexate [40mg/m² d1,8] + 5-Fluorouracil [600mg/m² d1,8]) × 6 postop
Austrian BCSG VII	N	(Cyclophosphamide [600mg/m² d1,8] + Methotrexate [40mg/m² d1,8] + 5-Fluorouracil [600mg/m² d1,8]) × 3 pre-op+ (Epirubicin [70mg/m² d1] + Cyclophosphamide [600mg/m² d1]) × 3 postop
	A	(Cyclophosphamide [600mg/m² d1,8] + Methotrexate [40mg/m² d1,8] + 5-Fluorouracil [600mg/m² d1,8]) × 3 postop + (Epirubicin [70mg/m² d1] + Cyclophosphamide [600mg/m² d1]) × 3 postop
EORTC 10902	N	5-Fluorouracil [600 mg/m² iv] + Epirubicin [60 mg/m² iv] + Cyclophosphamide [600 mg/m² iv]) q3wk × 4 pre-op
	A	5-Fluorouracil [600 mg/m² iv] + Epirubicin [60 mg/m² iv] + Cyclophosphamide [600 mg/m² iv]) q3wk × 4 postop
ECTO Italy	N	(Epirubicin [60mg/m² iv] then Paclitaxel [200mg/m² iv over 3h]) q3wk × 4 then (Cyclophosphamide [600mg/m² iv d1,8] + Methotrexate [40mg/m² iv d1,8] + 5-Fluorouracil [600mg/m² iv d1,8]) q4wk × 4 pre-op then Tam [20mg/d] to year 5
	A	(Epirubicin [60mg/m² iv] then Paclitaxel [200mg/m² iv over 3h]) q3wk × 4 then (Cyclophosphamide [600mg/m² iv d1,8] + Methotrexate [40mg/m² iv d1,8] + 5-Fluorouracil [600mg/m² iv d1,8]) q4wk × 4 postop then Tam [20mg/d] to year 5

Webtable 3: Definitions of tumour response and tumour response assessment within trial

Trial	Tumour response definition and method of assessment
BCCA Vancouver	Not measured, as only 1 cycle of preoperative chemotherapy was given
IB Bordeaux	cCR = complete disappearance of all evidence of tumour, PR = residual tumour size ≤2cm, SD/PD = residual tumour >2cm<br>Method: clinical examination and mammography. Pathological response not assessed.
Institut Curie S6	cCR = complete disappearance of all evidence of tumour, PR (major) = ≥50% reduction in initial tumour size, PR (minor) = 25-50% reduction, SD/PD undefined<br>Method: Clinical examination and mammography (size = greater of the two largest dimensions)
NSABP B-18	cCR = complete disappearance of all evidence of tumour, PR = ≥50% reduction in initial tumour size, SD = not CR/PR/PD , PD = ≥50% increase in tumour size<br>if cCR then, pCR=no histologic evidence of invasive tumour cells (non-invasive cells allowed), pINV=histologic evidence of invasive cells<br>Method: Clinical size quantified by the product of the two greatest tumour dimensions. Patients with complete clinical response, by physical examination, had their surgical specimens evaluated for pathologic tumour status.
St George's London	Employ UICC criteria (use callipers and mammogram). cCR = disappearance of all known disease, cPR = ≥50% reduction in initial tumour size,<br>NC = <50% reduction or <25% increase in size of lesion , PD = some lesions regress whilst others progress or new lesions appear.<br>Method: Tumour size measured with callipers by bi-dimensional measurements, multiplied to give area. Preliminary mammogram and a second 12 weeks before definitive surgery and radiotherapy.
RMH London	cCR = no palpable abnormality, cPR = ≥50% reduction in initial tumour size, NC = <50% reduction, PD = ≥25% increase in size<br>MRD = residual nodularity, classed within the PR category<br>Method: Palpation and tri-dimensional measurements of tumour size and blood flow using colour Doppler signals using an Acuson XP10 scanner. Mammography and ultrasound before surgery.
NCI Bethesda	Criteria from "The Breast Cancer Task Force Treatment Committee NCI. Publication 78-1192. DHEW, 1978. cCR = complete disappearance of all evidence of tumour, cPR = ≥50% reduction in initial tumour size , PD = ≥25% increase or new lesion, SD = not CR/PR/PD<br>Method: Palpation and mammography.
Austrian BCSG VII	pCR = complete disappearance of invasive cancer in final histological specimen of the primary tumour<br>PR = ≥50% reduction in initial tumour size, SD = <50% reduction, PD = any increase in tumour size<br>Method: Evaluated clinically, radiologically and histologically.
EORTC 10902	pCR = no signs of malignant cells at primary site or in lymph nodes, cCR = complete disappearance via palpation as well as mammography,<br>PR = ≥50% reduction in initial tumour size, PD = ≥25% increase in tumour size, or cN0 but developed palpable nodes<br>Method: Palpation and mammography. Product of the two greatest perpendicular diameters used to compare change in tumour size.
ECTO Italy	pCR = no residual invasive cancer in breast, irrespective of nodal status, cCR = no clinical evidence of disease (mammogram or ultrasound) or no new lesions,<br>cPR = ≥50% reduction in initial tumour size, cPR minor = ≥25% but <50% reduction, PD = ≥50% increase in tumour size or new lesion<br>Method: Physical examination. Mammography or ultrasound after chemotherapy completion. Surgical specimens evaluated for pathologic response.

Abbreviations: CR = complete response, PR = partial response, SD = stable disease, NC = no change, PD = progressive disease, c = clinical, p = pathological

Webtable 4: Participant characteristics, by trial

Characteristic	Category	BCCA Vancouver	IB Bordeaux	Institut Curie S6	NSABP B-18	St George's London	RMH London	NCI Bethesda	Austrian BCSG VII	EORTC 10902	ECTO Italy	Total
Age at entry (years)	<45	66	60	176	505	19	48	25	121	242	252	1514
	45-54	15	81	191	489	40	100	14	162	249	338	1679
	55+	0	131	23	529	44	157	14	146	207	312	1563
Clinical nodal status	Negative	47	0	161	1115	0	247	0	262	333	498	2663
	Positive	19	0	226	408	0	58	0	150	355	381	1597
	Unknown	15	272	3	0	103	0	53	17	10	23	496
Tumour size	1-19 mm	22	5	10	415	33	79	0	103	96	26	789
	20-49 mm	31	243	276	908	52	210	0	272	404	739	3135
	50+ mm	3	24	103	200	18	13	0	39	150	122	672
	Other/unknown	25	0	1	0	0	3	53	15	48	15	160
Biopsy grade	Well	0	39	0	0	0	0	0	0	0	88	127
	Moderate	6	145	0	0	0	0	0	0	0	482	633
	Poor	18	72	0	0	0	0	0	0	0	304	394
	Unknown	57	16	390	1523	103	305	53	429	698	28	3602
Biopsy ER status	ER-poor	34	161	152	0	103	0	0	364	122	340	1276
	ER-positive	40	111	192	0	0	0	0	42	313	548	1246
	Unknown	7	0	46	1523	0	305	53	23	263	14	2234
Biopsy PR status	PR-poor	7	161	156	0	0	0	0	332	0	383	1039
	PR-positive	14	110	187	0	0	0	0	66	0	501	878
	Unknown	60	1	47	1523	103	305	53	31	698	18	2839
Treatment allocation and clinical response	Neoadjuvant - Complete	0	30	0	240	17	27	0	25	23	184	546
	- Partial	0	32	0	304	14	93	0	104	148	108	803
	- Stable/progressive	0	64	0	140	21	23	0	49	144	157	598
	- Unknown	42	8	200	76	1	12	26	38	35	2	440
	Adjuvant only	39	138	190	763	50	150	27	213	348	451	2369
Planned local therapy	Radiotherapy only	0	0	390	0	0	0	0	0	0	0	390
	Lumpectomy	22	0	0	996	85	79	0	103	152	26	1463
	Mastectomy	3	272	0	527	18	13	0	39	536	122	1530
	Unknown	56	0	0	0	0	213	53	287	10	754	1373
**Total**	**All women**	**81**	**272**	**390**	**1523**	**103**	**305**	**53**	**429**	**698**	**902**	**4756**

ER = Oestrogen receptor, PR = Progesterone receptor

Webfigure 1: Rate ratios for effect of neo-adjuvant versus adjuvant chemotherapy on mortality by trial

Breast cancer mortality

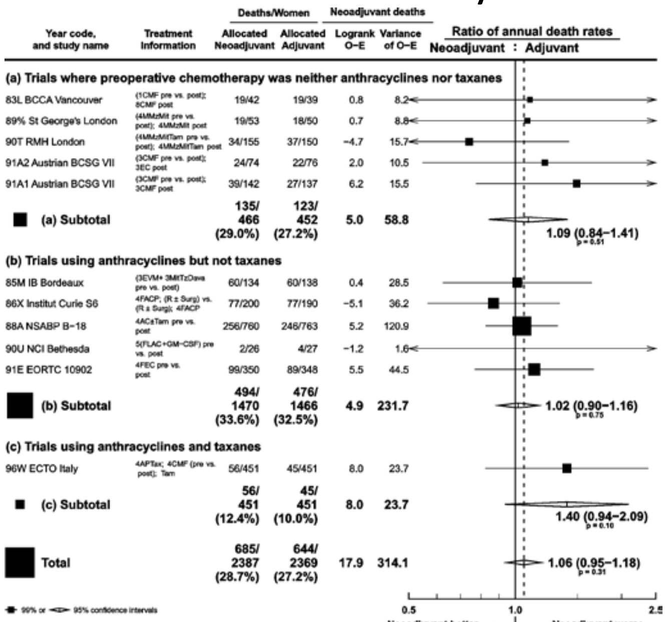

Heterogeneity between 3 subtotals: $\chi^2_2 = 2.2$; p = 0.33
Heterogeneity within subtotals: $\chi^2_8 = 6.4$; p = 0.60
Heterogeneity between 11 trials: $\chi^2_{10} = 8.6$; p = 0.57

Mortality without recurrence

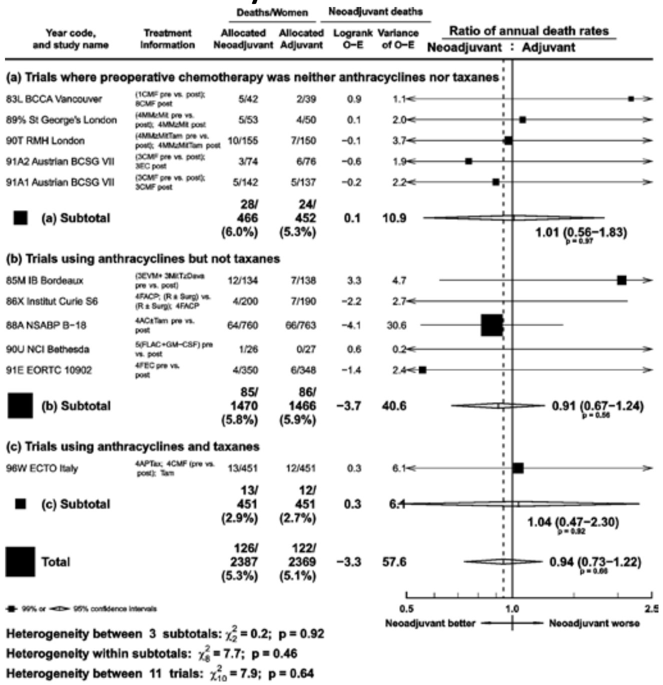

Heterogeneity between 3 subtotals: $\chi^2_2 = 0.2$; p = 0.92
Heterogeneity within subtotals: $\chi^2_8 = 7.7$; p = 0.46
Heterogeneity between 11 trials: $\chi^2_{10} = 7.9$; p = 0.64

Any death

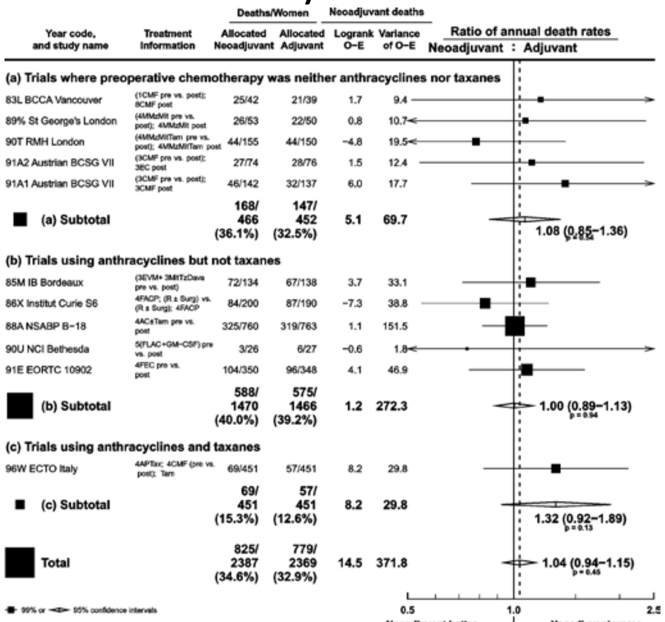

Heterogeneity between 3 subtotals: $\chi^2_2 = 2.1$; p = 0.35
Heterogeneity within subtotals: $\chi^2_8 = 5.7$; p = 0.68
Heterogeneity between 11 trials: $\chi^2_{10} = 7.8$; p = 0.65

Webtable 5: Breast tumour clinical response in women randomised to neo-adjuvant chemotherapy

Breast tumour clinical response*	Number of women	Percentage	
		All women (n=2387)	Women only with known response (n=1947)
Complete (CR)	546	23	28
Partial (PR)	803	34	41
Stable/progressive disease (SD/PD)	598	25	31
Unknown	440	18	-
**Total**	**2387**	**100**	**100**

*CR = no clinical evidence of disease, PR = ≥50% reduction in initial tumour size, SD/PD = <50% reduction, or increase in size.

Information on individual responses was not available from the Institut Curie S6 and NCI Bethesda trials, but publications from those two trials have reported the numbers of women with CR/PR/SD or PD/Unk to be 46/80/27/47 and 11/2/4/9 respectively.

BCCA Vancouver gave only one cycle of neo-adjuvant chemotherapy, and did not record the clinical response to it.

Webtable 6: Planned surgery versus surgery actually undertaken, by trial and allocated treatment

Trial	Surgery undertaken	Planned surgery									
		Neo-adjuvant					Adjuvant				
		**RT only**	**Lx**	**Mx**	**Unk**	**Total**	**RT only**	**Lx**	**Mx**	**Unk**	**Total**
BCCA Vancouver	No surgery				1	**1**		1			**1**
	Lumpectomy		6		7	**13**		3		9	**12**
	Mastectomy		7		21	**28**		5	3	18	**26**
IB Bordeaux	No surgery			30		**30**					
	Lumpectomy			32		**32**					
	Mastectomy			64		**64**			138		**138**
	Unknown			8		**8**					
Institut Curie S6	Lumpectomy*	167				**167**	144				**144**
	Mastectomy	33				**33**	46				**46**
NSABP B-18	No surgery		6	2		**8**		1	4		**5**
	Lumpectomy		440	70		**510**		419	35		**454**
	Mastectomy		50	192		**242**		80	223		**303**
	Unknown								1		**1**
St George's London	No surgery		7	3		**10**		1	1		**2**
	Lumpectomy		31	4		**35**		41	1		**42**
	Mastectomy		5	3		**8**			6		**6**
RMH London	Lumpectomy		34	5	98	**137**		40	4	72	**116**
	Mastectomy		2	1	13	**16**		3	3	26	**32**
	Unknown				2	**2**				2	**2**
NCI Bethesda	Unknown				26	**26**				27	**27**
Austrian BCSG VII	Lumpectomy		36	4	93	**133**		39	2	78	**119**
	Mastectomy		10	14	41	**65**		14	15	54	**83**
	Unknown		2	3	13	**18**		2	1	8	**11**
EORTC 10902	No surgery			20	3	**23**		1	9	6	**16**
	Lumpectomy		63	57	1	**121**		64	13		**77**
	Mastectomy		15	191		**206**		9	246		**255**
ECTO Italy	Lumpectomy		11	25	248	**284**		13	4	130	**147**
	Mastectomy		1	37	116	**154**		1	50	243	**294**
	Unknown			3	10	**13**			3	7	**10**
All trials	No Surgery		13	55	4	**72**		4	14	6	**24**
	Lumpectomy	167	621	197	447	**1432**	144	619	59	289	**1111**
	Mastectomy	33	90	502	191	**816**	46	112	684	341	**1183**
	Unknown		2	14	51	**67**		2	5	44	**51**
	**Total**	**200**	**726**	**768**	**693**	**2387**	**190**	**737**	**762**	**680**	**2369**

Lx = Lumpectomy, Mx = Mastectomy, RT = Radiotherapy

In the adjuvant arms, 80% with clinical tumour size <2 cm had lumpectomy and 80% with tumour size ≥5 cm had mastectomy. Hence, if the planned surgery was unknown, it was taken as lumpectomy if size was <2 cm and mastectomy if size was ≥5 cm. This convention was used to re-categorise 408 women. (Clinical nodal status was not a good predictor of planned surgery.)

*In the Institut Curie S6 trial, lumpectomy includes "No surgery", and information on each individual was not available. Publications from this trial have reported RT only 102 neo-adjuvant vs 87 adjuvant and BCS+RT 62 neo-adjuvant vs 60 adjuvant.

Webfigure 2: Planned surgery versus surgery actually undertaken, by allocated treatment group

A) Excluding trials IB Bordeaux and Institut Curie S6

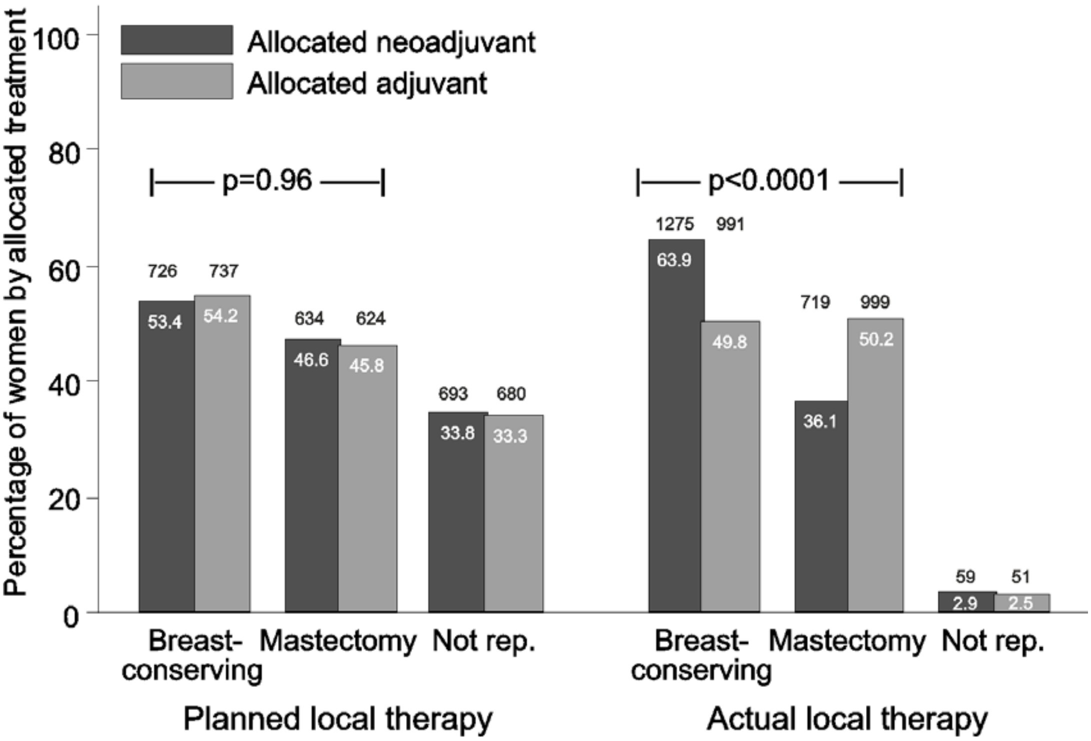

B) Including all trials

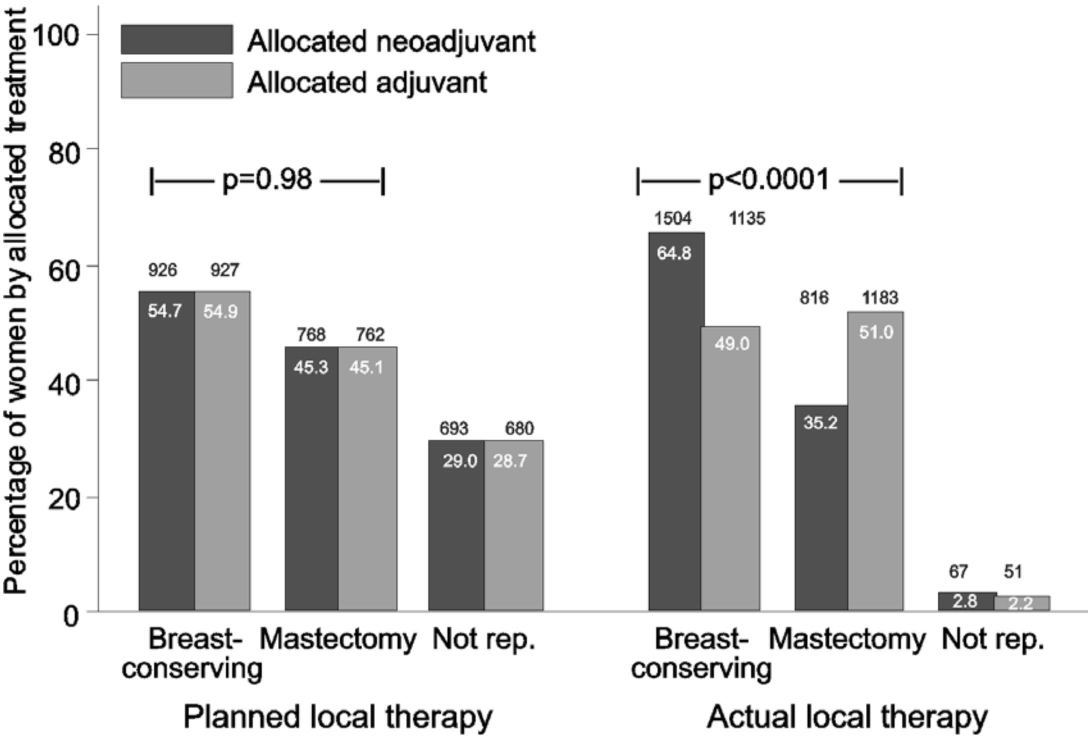

Percentages for breast-conserving and mastectomy exclude unknowns. Percentages for unknowns show the fraction of the total number of patients, in the respective allocated treatment groups, with missing local therapy information.

Webtable 7: Local recurrence in women randomised to neo-adjuvant chemotherapy by their sequence of planned and actual local therapy.

For women randomised to neo-adjuvant chemotherapy, the table shows the *crude* 10-year cumulative risk of local recurrence according to their combination of planned surgery (ie decision before any chemotherapy) and the surgery they actually received (after chemotherapy). It also shows the hazard ratios for local recurrence for these women by surgery sequence, unadjusted and adjusted for clinical tumour size and stratified by trial.

Planned/ Actual Surgery e	Unadjusted local recurrence risk % at year 10 (95% CI)	Unadjusted hazard ratio (HR)*		Adjusted hazard ratio (HR)*	
		HR	95% CI	HR	95% CI
Mx/BCT (n=252)	23.4 (17.8, 30.2)	1.00	(0.76, 1.32)	1.00	(0.73, 1.36)
Mx/Mx (n=502)	19.8 (15.7, 24.8)	0.88	(0.70, 1.11)	0.97	(0.75, 1.25)
BCT/BCT (n=801)	21.2 (18.2, 24.6)	0.96	(0.83, 1.12)	0.84	(0.65, 1.10)
BCT/Mx (n=123)	13.7 (8.2, 22.4)	0.52	(0.31, 0.88)	0.39	(0.22, 0.68)
P for heterogeneity		0.09		0.01	

BCT = breast-conserving therapy (ie radiotherapy alone or lumpectomy with or without radiotherapy), Mx = Mastectomy.

Planned surgery is labelled first and actual surgery last ie, planned/actual. Planned surgery may differ from actual surgery if the response of the breast tumour to neo-adjuvant chemotherapy leads to de-escalation of the planned extent of local therapy.

* HR = hazard ratio for the planned/actual surgery group relative to the Mx/BCT group, derived from a Cox regression either unadjusted, or adjusted for clinical tumour size and stratified by trial. Confidence intervals are group specific (Plummer 2004).

Reference

Plummer M**.** Improved estimates of floating absolute risk. *Stat Med* 2004; **23**(1): 93-104.

Webfigure 3: Rate ratios for effect of neo-adjuvant versus adjuvant chemotherapy on local recurrence by extent of planned local therapy and trial

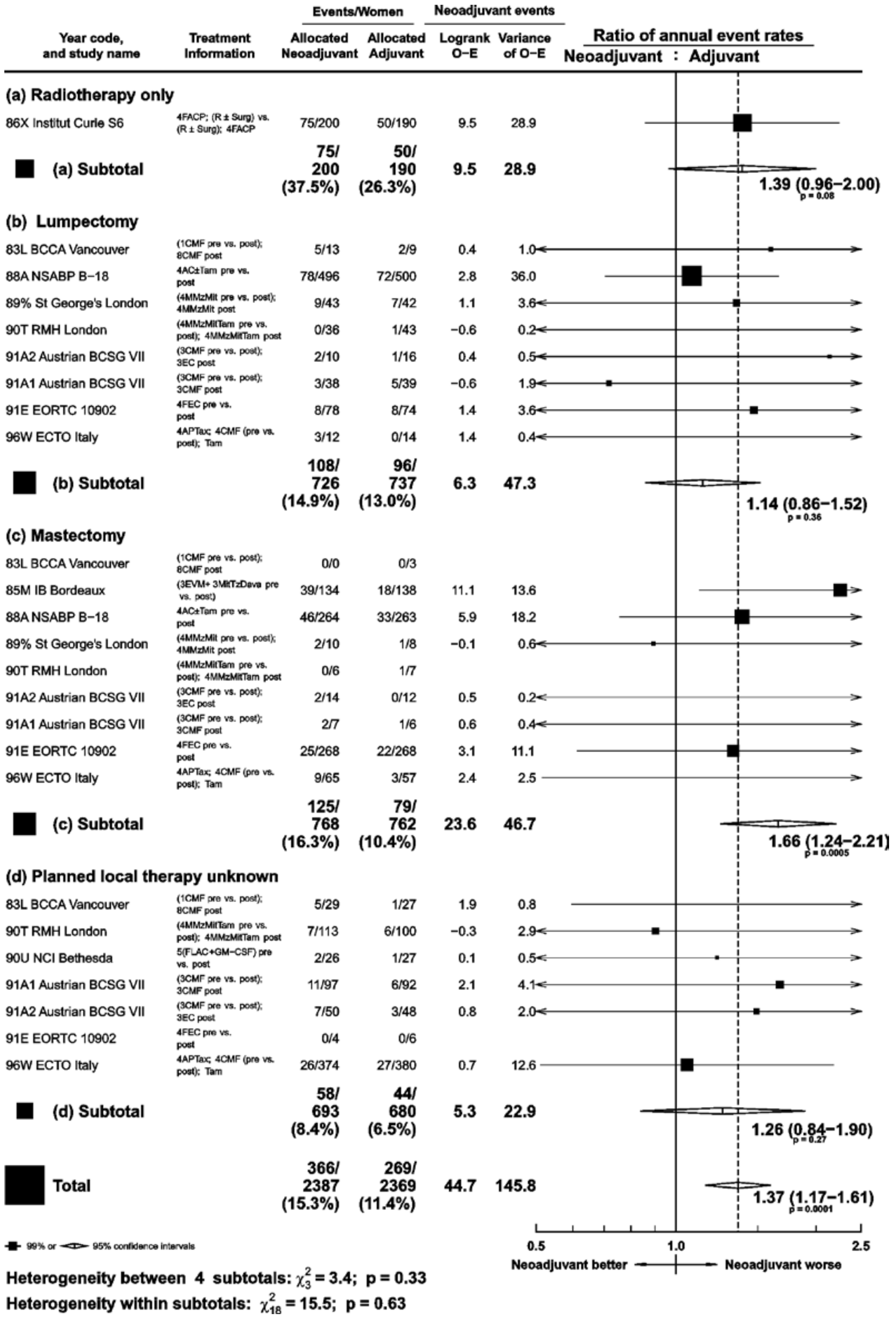

Abbreviations CMF, cyclophosphamide, methotrexate, 5-fluorouracil; MMzMit, methotrexate, mitoxantrone, mitomycin-C; EVM/MitTzDAVA, epirubicin, vincristine, methotrexate/Mitomycin-C, triethylenephosphoramide, vindesine; FAC, fluorouracil, doxorubicin, cyclophosphamide; AC, doxorubicin, cyclophosphamide; Tam, tamoxifen; FLAC, fluorouracil, leucovorin, doxorubicin, cyclophosphamide; GM-CSF, granulocyte-macrophage colony-stimulating factor; FEC, fluorouracil, epirubicin, cyclophosphamide; APTax, doxorubicin, paclitaxel.

See Webtable 2 for a full description of each chemotherapy regimen.

Webfigure 4: Rate ratios for effect of neo-adjuvant versus adjuvant chemotherapy on recurrence and breast cancer mortality by various factors

Local recurrence

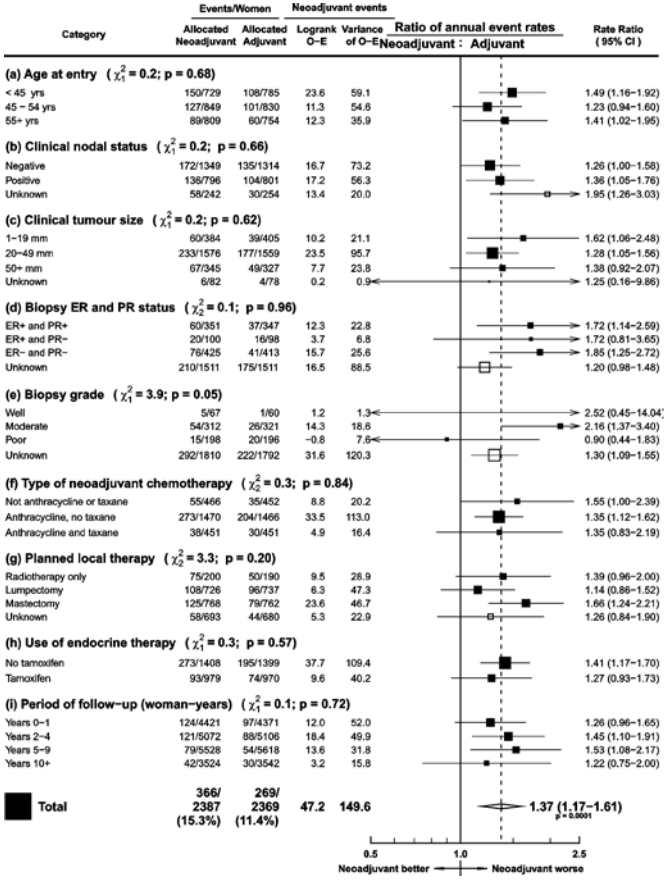

Distant recurrence*

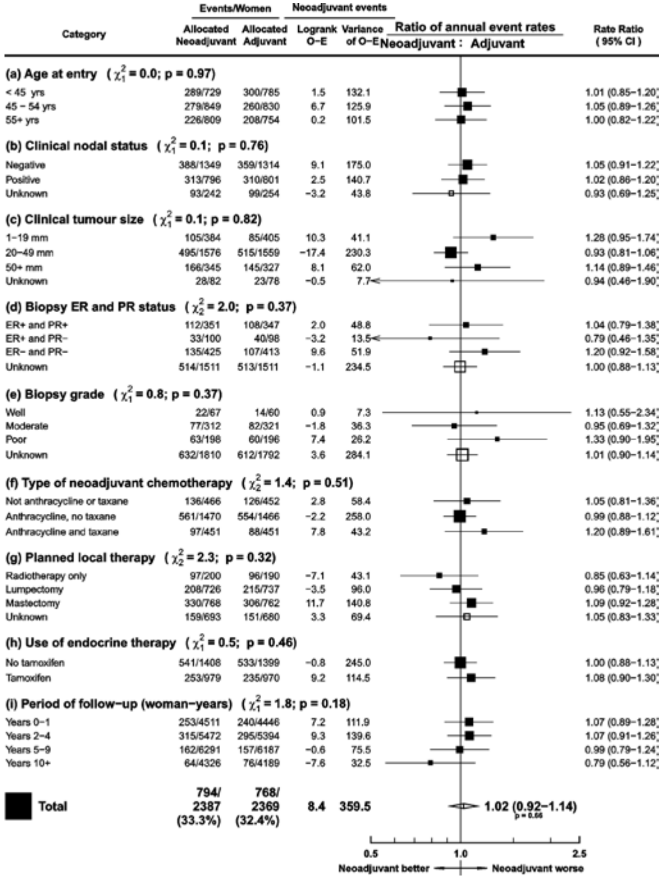

Breast cancer mortality

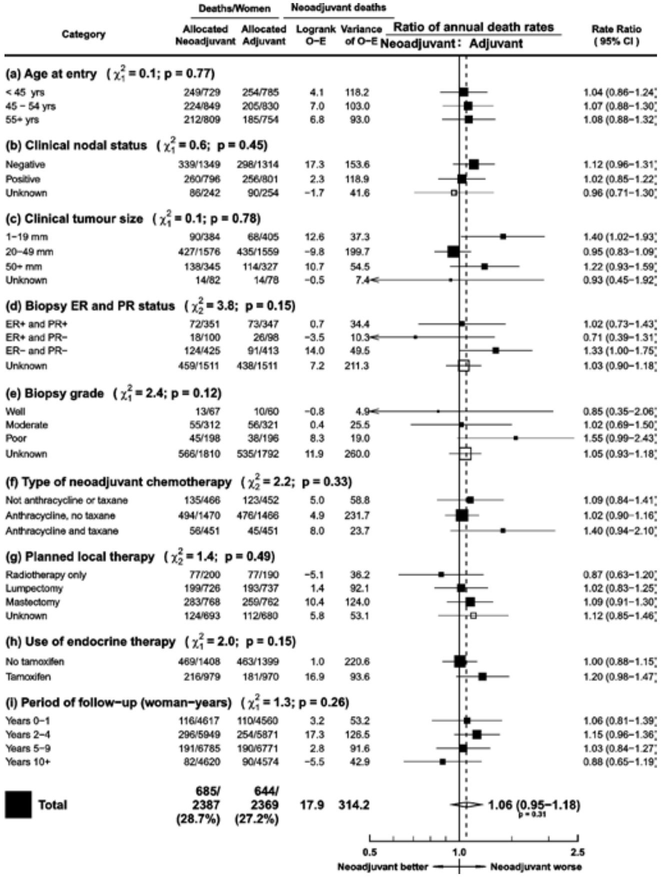

* NSABP B-18, NCI Bethesda, and Austrian BCSG VII recorded only the first event, so distant recurrence means either distant recurrence as first event or death from breast cancer.

Webfigure 5: Comparison of women who had either a complete, partial or no clinical response to neo-adjuvant chemotherapy to all women randomised to adjuvant chemotherapy for recurrence and mortality (largely reflects patient selection rather than treatment effect)

Local recurrence Distant recurrence* Breast cancer mortality

546 complete clinical responders are compared to all women randomised to adjuvant chemotherapy

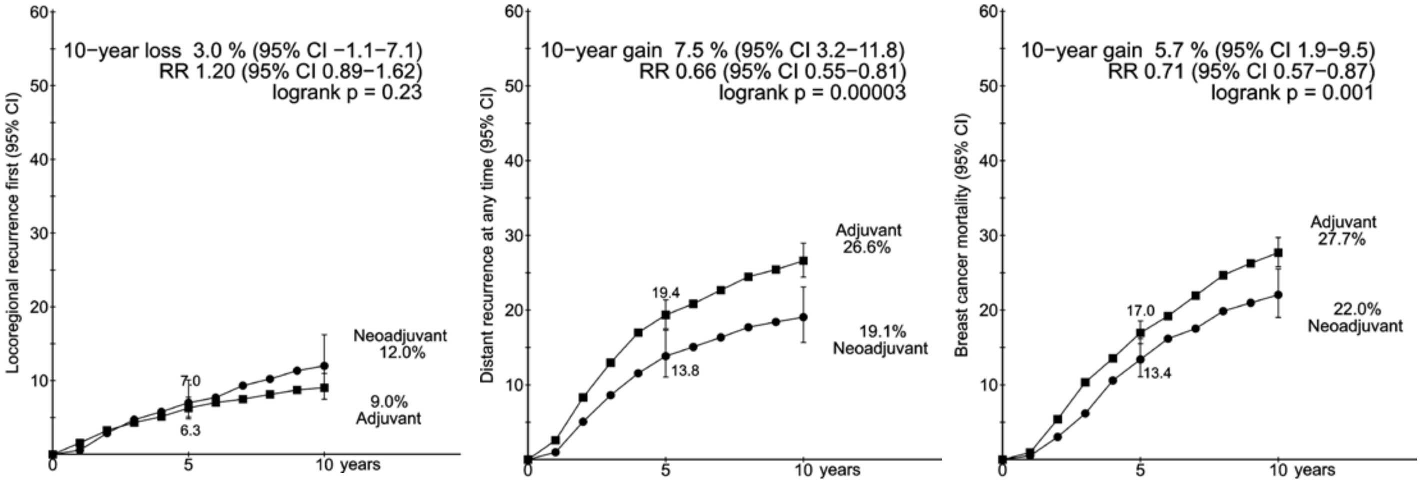

803 partial clinical responders are compared to all women randomised to adjuvant chemotherapy

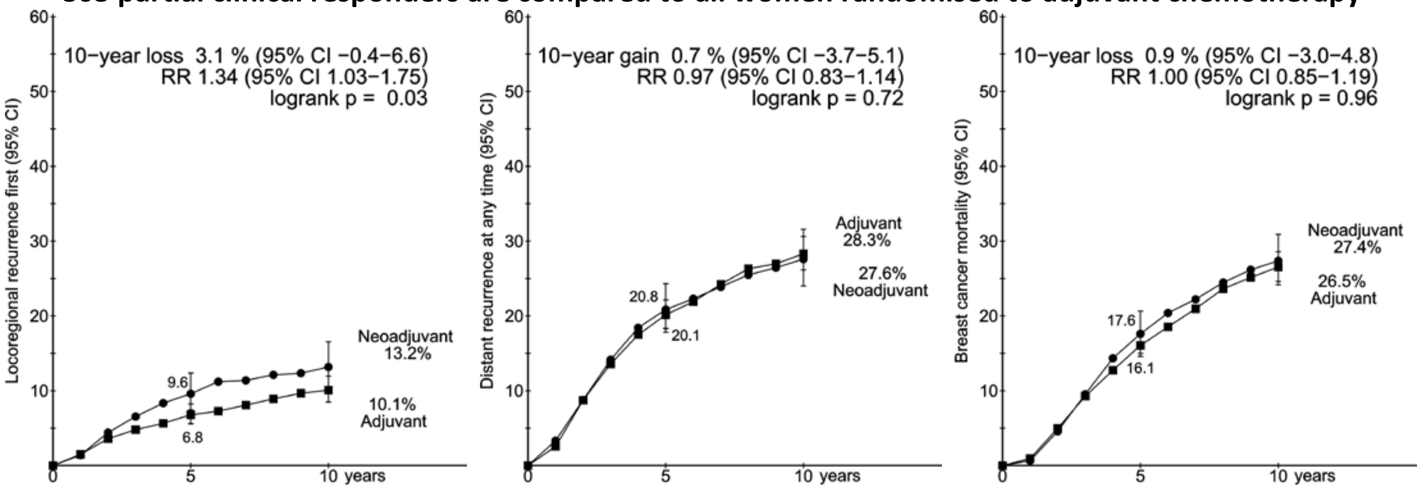

598 clinical non-responders are compared to all women randomised to adjuvant chemotherapy

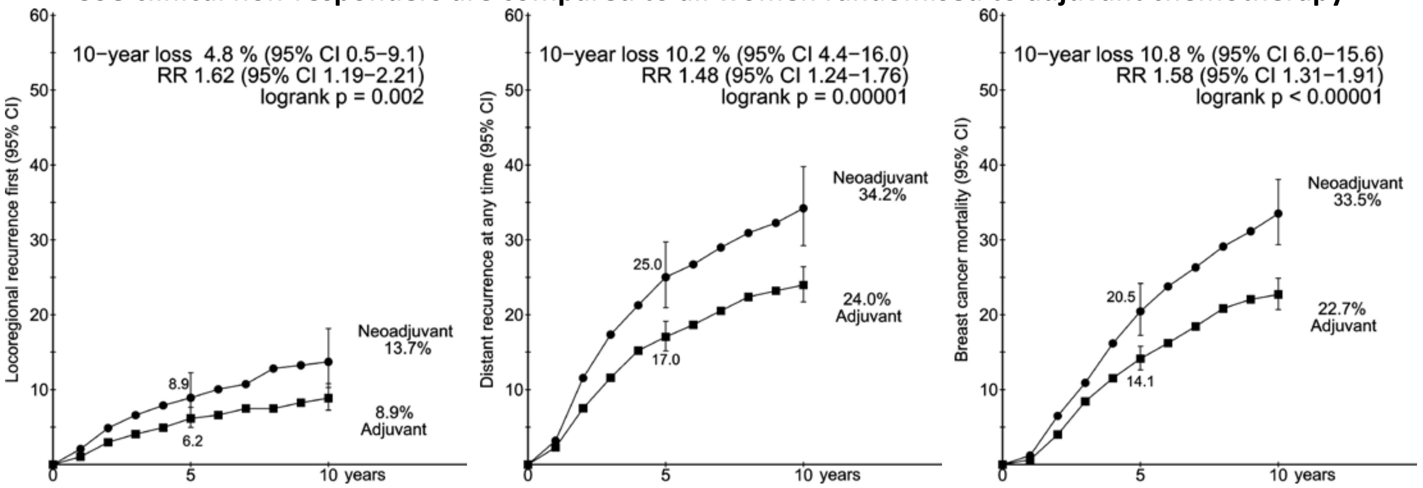

Analyses stratified by trial, follow-up year, age at entry, pre-randomisation clinical nodal status and tumour size. BCCA Vancouver, Institut Curie S6 and NCI Bethesda trials are excluded because individual breast responses were not available. Complete response: no clinical evidence of disease, partial response: ≥50% reduction in initial tumour size, non-response: <50% reduction or increase.
*NSABP B-18 and Austrian BCSG VII recorded only first events, so distant recurrence means as first event, or breast cancer death.

Webfigure 6: Percentage complete clinical response (CR) by trial and breast-conserving therapy rate

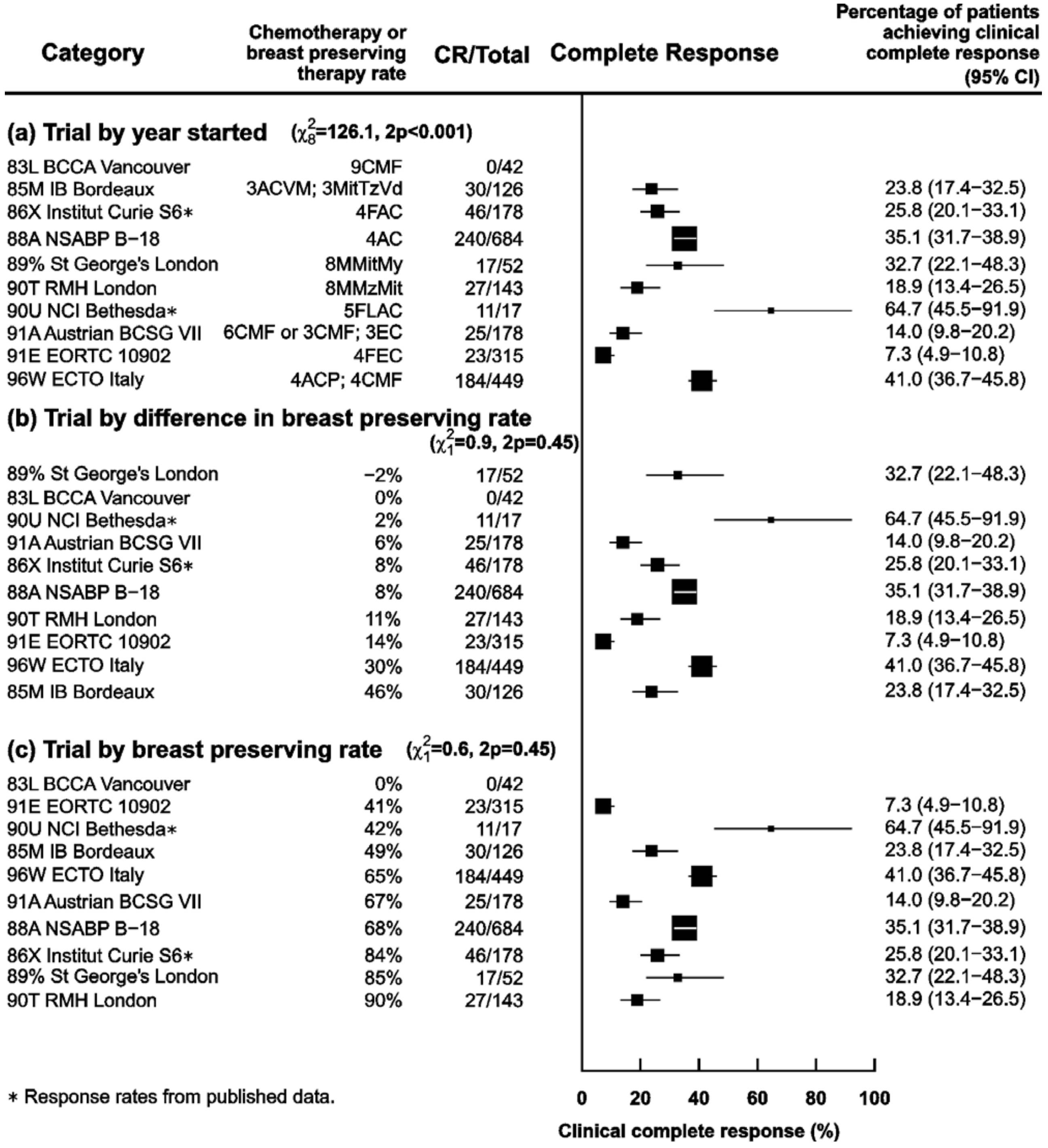

As BCCA Vancouver gave only course of CMF pre-operatively, it is assumed here that CR=0.

Category (b): Difference is between the breast-conserving rate in the adjuvant-only and the neo-adjuvant arms.

Webfigure 7: Rate ratios for effect of neo-adjuvant versus adjuvant chemotherapy on recurrence and breast cancer mortality by trial and by percentage of women with breast tumour complete clinical response and breast-conserving rate

Local recurrence

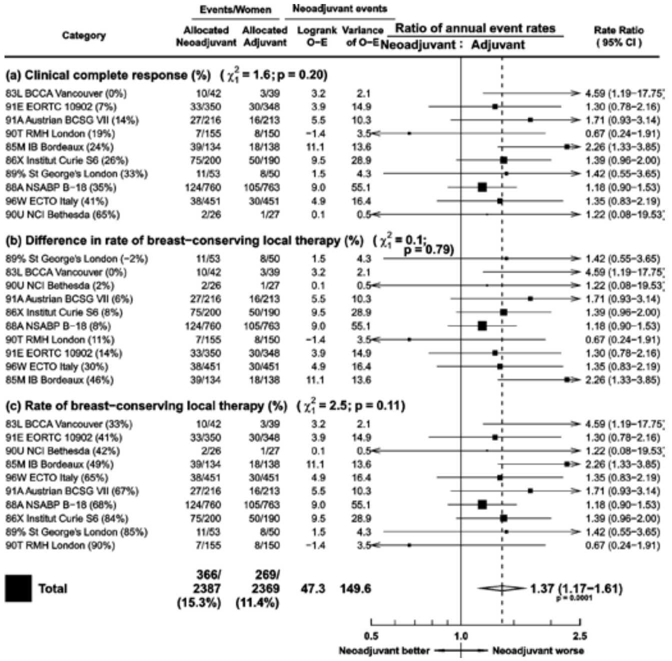

Distant recurrence*

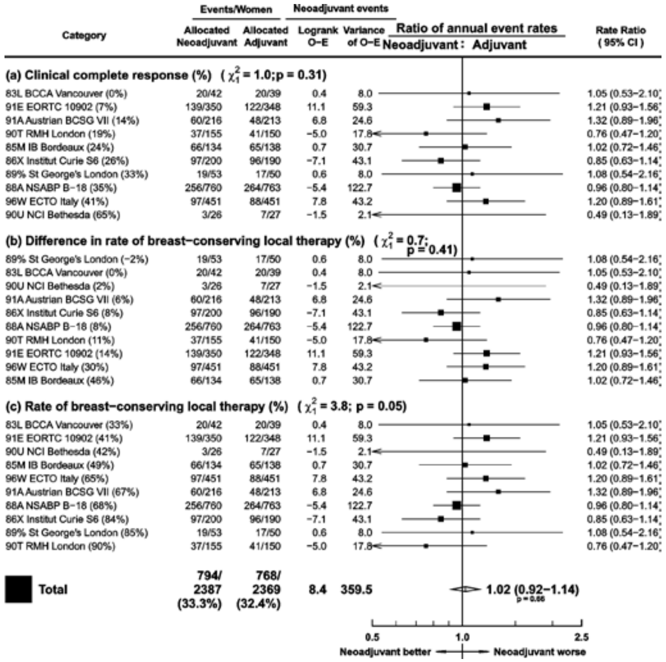

Breast cancer mortality

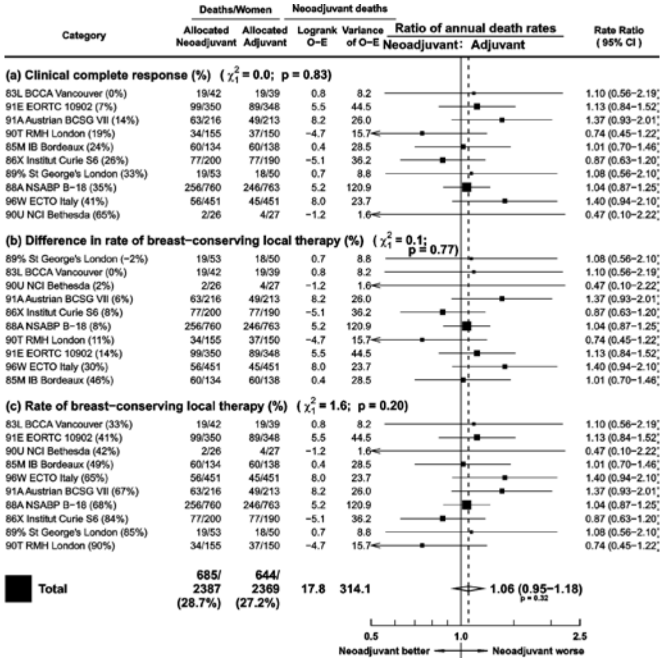

As BCCA Vancouver gave only course of CMF pre-operatively, it is assumed here that CR=0.

Tumour response and breast-conserving rates not available as individual data for all trials and therefore are taken from trial publications (Institut Curie S6 and NCI Bethesda).

*NSABP B-18 and Austrian BCSG VII recorded only first events, so distant recurrence means either distant recurrence as first event, or breast cancer death.

Webtable 8: Eligible randomised trials identified but not available for analysis; total ~500 patients

Trial **Institut Curie S5 - France (1983-1986)**
Description 196 patients entered; trial terminated early, with much incomplete or omitted treatment.
Arms (Doxorubicin [25mg/m^2 d1,8]+Cyclophosphamide [400mg/m^2 d1,8]+5-Fluorouracil [500mg/m^2 d1,3,5,8]) q4w ×2 pre-op
(Doxorubicin [25mg/m^2 d1,8]+Cyclophosphamide [400mg/m^2 d1,8]+5-Fluorouracil [500mg/m^2 d1,3,5,8]) q4w ×4 postop
vs
(Doxorubicin [25mg/m^2 d1,8]+Cyclophosphamide [400mg/m^2 d1,8]+5-Fluorouracil [500mg/m^2 d1,3,5,8]) q4w ×6 postop
Reference *Scholl SM, Asselain B, Palangie T et al. Neoadjuvant chemotherapy in operable breast cancer.*
*Eur J Ca 1991; **27:** 1668-71.*

Trial **All-India Institute of Medical Sciences, New Delhi - India (1997-2001)**
Description 101 patients entered; all received radiotherapy
Arms (Cyclophosphamide [500mg/m^2 d1,15]+Epirubicin [50mg/m^2 d1,15]+5-Fluorouracil [500mg/m^2 d1,15])q4wk ×3 pre-op
(Cyclophosphamide [500mg/m^2 d1,15]+Epirubicin [50mg/m^2 d1,15]+5-Fluorouracil [500mg/m^2 d1,15])q4wk ×3 postop
vs
(Cyclophosphamide [500mg/m^2 d1,15]+Epirubicin [50mg/m^2 d1,15]+5-Fluorouracil [500mg/m^2 d1,15])q4wk ×6 postop
Reference *Deo SVS, Bhutani, M, Shukla, NK, Raina V, Rath GK, Purkayasth J. Randomized trial comparing neo-adjuvant versus adjuvant chemotherapy in operable locally advanced breast cancer (T4b N0-2 M0). J Surg Oncol 2003; **84**(4): 192-97.*

Trial **Shanghai - China (pre-1990)**
Description 57 patients included; all received excisional biopsy followed by either radiotherapy or mastectomy.
Arms (Cyclophosphamide [600mg/m^2 d1,8]+Methotrexate [30mg/m^2 d1,8]+5-Fluorouracil [500mg/m^2 d1,8]) ×2 pre-op
(Cyclophosphamide [600mg/m^2 d1,8]+Methotrexate [30mg/m^2 d1,8]+5-Fluorouracil [500mg/m^2 d1,8]) ×2 postop
vs Placebo [d1,8] × 2 pre-op
(Cyclophosphamide [600mg/m^2 d1,8]+Methotrexate [30mg/m^2 d1,8]+5-Fluorouracil [500mg/m^2 d1,8]) ×6 postop
Reference *Shao ZM, Li J, Wu J, et al. Neo-adjuvant chemotherapy for operable breast cancer induces apoptosis.*
*Breast Ca Res Treat 1999; **53**(3): 263-69.*

Trial **Huguenin Chemotherapy - France (1989)**
Description <40 patients included; trial abandoned early
Arms (5-Fluorouracil [500mg/m^2]+Doxorubicin [50mg/m^2]+Cyclophosphamide [500mg/m^2]) ×4 pre-op
(5-Fluorouracil [500mg/m^2]+Doxorubicin [50mg/m^2]+Cyclophosphamide [500mg/m^2]) ×2 postop
vs
(5-Fluorouracil [500mg/m^2]+Doxorubicin [50mg/m^2]+Cyclophosphamide [500mg/m^2]) ×6 postop
Reference *Tubiana-Hulin M, Kerbrat P. Trial protocol: Chemiothérapie première des cancers du sein opérables. Unpublished, 1989.*

Trial **Tokyo - Japan (1995-1997)**
Description 50 patients included; all had mastectomy, but some were excluded after randomization.
Arms (Epirubicin [50mg/m^2]+Cyclophosphamide [200mg/m^2])q3wk ×5 pre-op
vs
(Epirubicin [50mg/m^2]+Cyclophosphamide [200mg/m^2])q3wk ×5 postop
Reference *Enomoto K, Ikeda T, Matsui A, et al. Neoadjuvant therapy in stage II with T>=4CM and stage III breast cancer.*
6th International Conference on adjuvant therapy for primary breast cancer, St Gallen 1998: Poster P73.

Trial **French Adjuvant Study Group - France (GFEA 04) (1990)**
Description Estimated 50 patients included in a 4-arm study, in which only the last 2 of the 4 arms would have been relevant.
Arms (Epirubicin+5-Fluorouracil+Cyclophosphamide) ×3 pre-op
(Epirubicin+5-Fluorouracil+Cyclophosphamide) ×3 postop
(Epirubicin+5-Fluorouracil+Cyclophosphamide) ×3 pre-op; (Epirubicin+5-Fluorouracil+Cyclophosphamide) ×3 postop
(Epirubicin+5-Fluorouracil+Cyclophosphamide) ×6 postop
Reference *No reference available.*

Webtable 9: Sensitivity analyses of using different methods to assess the effect of neo-adjuvant chemotherapy on local and distant recurrence

A. Effect of ignoring competing events (distant recurrence/non-breast death) on local recurrence rate ratio

The local recurrence rate ratio from logrank methods was compared with that from Cox regression (using the same stratification as in the logrank method) and from a competing risks model using Cox regression. The local recurrence rate ratio varied little with the statistical method used.

Statistical method	Local recurrence rate ratio (95% CI)
Logrank	1.37 (1.17-1.61)
Cox regression	1.37 (1.17-1.61)
Competing risks model	1.41 (1.20-1.64)

Reference: Fine JP, Gray RJ. A proportional hazards model for the sub-distribution of a competing risk. *JASA* 1999; **94**: 496-509.

B. Distant recurrence and recording of only the first event or of first and subsequent events

Some trials collected only first events, but some recorded first and subsequent events. Analyses of distant recurrence in the two types of trial are compared. Rate ratios and failure rates varied little between them.

Events recorded	Number of women	Rate ratio (95% CI) for distant recurrence	Percentage with distant recurrence (Neoadjuvant vs Adjuvant only)	
			10 years after randomisation	15 years after randomisation
First events and subsequent events	2751	1.04 (0.91-1.19)	36.2% vs 33.9%	40.8% vs 39.5%
Only first events	2005	1.00 (0.85-1.17)	30.8% vs 30.5%	35.3% vs 36.2%
**All trials**	**4756**	**1.02 (0.92-1.14)**	**33.8% vs 32.4%**	**38.2% vs 38.0%**

Webfigure 8: EBCTCG collaborators, alphabetically by institution or study then alphabetically by name

AARTM 048/13/2000 Multicentre Study Group, Spain—J A Alberro, B Ballester, P Deulofeu, R Fábregas, M Fraile, J M Gubern, J Janer, A Moral, J L de Pablo, G Peñalva, P Puig, M Ramos, R Rojo, P Santesteban, C Serra, M Solà, L Solarnau, J Solsona, E Veloso, S Vidal.
ACETBC, Tokyo, Japan—O Abe, R Abe, K Enomoto, K Kikuchi, H Koyama, H Masuda, Y Nomura, Y Ohashi, K Sakai, K Sugimachi, M Toi, T Tominaga, J Uchino, M Yoshida.
Addenbrooke's Hospital, Cambridge, UK—C E Coles, J L Haybittle.
AGO Breast Study Group (AGO-B), Germany—V Möbus.
Anglo-Celtic Cooperative Oncology Group, UK—C F Leonard.
ARCOSEIN Group, France—G Calais, P Garaud.
ATLAS Trial Collaborative Study Group, Oxford, UK—V Collett, C Davies, A Delmestri, J Sayer.
Auckland Breast Cancer Study Group, New Zealand—V J Harvey, I M Holdaway, R G Kay, B H Mason.
Australian New Zealand Breast Cancer Trials Group, Sydney, Australia—J F Forbes, P A Francis, N Wilcken.
Austrian Breast Cancer Study Group, Vienna, Austria—M Balic, R Bartsch, C Fesl, F Fitzal, H Fohler, M Gnant, R Greil, R Jakesz, C Marth, B Mlineritsch, G Pfeiler, C F Singer, G G Steger, H Stöger.
Beatson Oncology Centre, Glasgow, UK—P Canney, H M A Yosef.
Belgian Adjuvant Breast Cancer Project, Liège, Belgium—C Focan.
Berlin-Buch Akademie der Wissenschaften, Germany—U Peek.
Birmingham General Hospital, UK—G D Oates, J Powell.
Bordeaux Institut Bergonié, France—M Durand, L Mauriac.
Bordet Institute, Brussels, Belgium—A Di Leo, S Dolci, D Larsimont, J M Nogaret, C Philippson, M J Piccart.
Bradford Royal Infirmary, UK—M B Masood, D Parker, J J Price.
Breast Cancer International Research Group (BCIRG)—M A Lindsay, J Mackey, M Martin.
Breast Cancer Study Group of the Comprehensive Cancer Centre, Limburg, Netherlands—P S G J Hupperets.
British Association of Surgical Oncology BASO II Trialists, London, UK—T Bates, R W Blamey, U Chetty, I O Ellis, E Mallon, D A L Morgan, J Patnick, S Pinder.
British Columbia Cancer Agency, Vancouver, Canada—C Lohrisch, A Nichol.
Canadian Cancer Trials Group, Kingston, Ontario, Canada—J M S Bartlett, V H Bramwell, B E Chen, S K L Chia, K Gelmon, P E Goss, M N Levine, W Parulekar, J L Pater, K I Pritchard, L E Shepherd, D Tu, T Whelan.
Cancer and Leukemia Group B, Washington DC, USA—D Berry, G Broadwater, C Cirrincione, H Muss, L Norton, R B Weiss.
Cancer Care Ontario, Canada—H T Abu-Zahra.
Cancer Research Centre of the Russian Academy of Medical Sciences, Moscow, Russia—A Karpov, S M Portnoj.
Cancer Research UK Clinical Trials Unit (CRCTU), NCRI, Birmingham, UK—S Bowden, C Brookes, J Dunn, I Fernando, M Lee, C Poole, D Rea, D Spooner.
Cardiff Trialists Group, UK—P J Barrett-Lee, R E Mansel, I J Monypenny.
Case Western Reserve University, Cleveland, OH, USA—N H Gordon.
Central Oncology Group, Milwaukee, WI, USA—H L Davis.
Centre for Cancer Prevention, Wolfson Institute of Preventive Medicine, Queen Mary, University of London, UK—J Cuzick, I Sestak.
Centre Léon-Bérard, Lyon, France—Y Lehingue, P Romestaing.
Centre Paul Lamarque, Montpellier, France—J B Dubois.
Centre Regional François Baclesse, Caen, France—T Delozier, B Griffon, J Mace Lesec'h.
Centro Oncologico, Trieste, Italy—G Mustacchi.
Charles University in Prague, First Faculty of Medicine, Department of Oncology of the First Faculty of Medicine and General Teaching Hospital, Czech Republic—L Petruzelka, O Pribylova.
Cheltenham General Hospital, UK—J R Owen.
Chemo N0 Trial Group, Germany—N Harbeck, F Jänicke, C Meisner, M Schmitt, C Thomssen.

Chicago University, IL, USA—P Meier.
Chinese Academy of Medical Sciences, Beijing, People's Republic of China (in collaboration with the Oxford MRC PHRU)—Y Shan, Y F Shao, X Wang, D B Zhao (MRC PHRU: Z M Chen, H C Pan).
Christie Hospital and Holt Radium Institute, Manchester, UK—A Howell, R Swindell.
Coimbra Instituto de Oncologia, Portugal—J Albano, C F de Oliveira, H Gervásio, J Gordilho.
Copenhagen Breast Cancer Trials, Copenhagen, Denmark—B Ejlertsen, M-B Jensen, H Mouridsen.
Dana-Farber Cancer Institute, Boston, MA, USA—R S Gelman, J R Harris, D Hayes, C Henderson, C L Shapiro, E Winer.
Danish Breast Cancer Cooperative Group, Copenhagen, Denmark— P Christiansen, B Ejlertsen, M Ewertz, M-B Jensen, H T Mouridsen.
Düsseldorf University, Germany—T Fehm, H J Trampisch.
Dutch Working Party for Autologous Bone Marrow Transplant in Solid Tumours, Amsterdam & Groningen, Netherlands—O Dalesio, E G E de Vries, S Rodenhuis, H van Tinteren.
Eastern Cooperative Oncology Group, Boston, MA, USA—R L Comis, N E Davidson, R Gray, N Robert, G Sledge, L J Solin, J A Sparano, D C Tormey, W Wood.
Edinburgh Breast Unit, UK—D Cameron, U Chetty, J M Dixon, P Forrest, W Jack, I Kunkler.
Elim Hospital, Hamburg, Germany—J Rossbach.
Erasmus MC/Daniel den Hoed Cancer Center, Rotterdam, Netherlands— J G M Klijn, A D Treurniet-Donker, W L J van Putten.
European Institute of Oncology, Milan, Italy—N Rotmensz, U Veronesi, G Viale.
European Organization for Research and Treatment of Cancer, Brussels, Belgium—H Bartelink, N Bijker, J Bogaerts, F Cardoso, T Cufer, J P Julien, P M Poortmans, E Rutgers, C J H van de Velde.
Evanston Hospital, IL, USA—M P Cunningham.
Finnish Breast Cancer Group, Finland—R Huovinen, H Joensuu.
Fondazione Maugeri Pavia, Italy—A Costa.
Fondazione Michelangelo, Milan, Italy—G Bonadonna, L Gianni, P Valagussa.
Fox Chase Cancer Center, Philadelphia, PA, USA—L J Goldstein.
French Adjuvant Study Group (GFEA), Guyancourt, France—J Bonneterre, P Fargeot, P Fumoleau, P Kerbrat, E Luporsi, M Namer.
GEICAM, Spanish Breast Cancer Group, Spain—E Carrasco, M Martin, M A Segui.
German Adjuvant Breast Group (GABG), Frankfurt, Germany—W Eiermann, J Hilfrich, W Jonat, M Kaufmann, R Kreienberg, M Schumacher.
German Breast Cancer Study Group (BMFT), Freiburg, Germany—G Bastert, H Rauschecker, R Sauer, W Sauerbrei, A Schauer, M Schumacher.
German Breast Group (GBG), Neu-Isenburg, Germany—J U Blohmer, S D Costa, H Eidtmann, B Gerber, C Jackisch, S Loibl, G von Minckwitz.
Ghent University Hospital, Belgium—A de Schryver, L Vakaet.
GIVIO Interdisciplinary Group for Cancer Care Evaluation, Chieti, Italy—M Belfiglio, A Nicolucci, F Pellegrini, M C Pirozzoli, M Sacco, M Valentini.
Glasgow Victoria Infirmary, UK—C S McArdle, D C Smith, S Stallard.
Groote Schuur Hospital, Cape Town, South Africa—D M Dent, C A Gudgeon, A Hacking, E Murray, E Panieri, ID Werner.
Gruppo Interdisciplinare Veneto di Oncologia Mammaria (GIVOM), Italy—G L De Salvo, P Del Bianco, G Zavagno.
Grupo Oncológico Cooperativo del Sur (GOCS), Argentina—B Leone, C T Vallejo, A Zwenger.
Gruppo Oncologico Clinico Cooperativo del Nord Est, Aviano, Italy—E Galligioni.
Gruppo Oncologico Dell'Italia Meridionale (GOIM), Rome, Italy—M Lopez.
Guadalajara Hospital de 20 Noviembre, Mexico—A Erazo, J Y Medina.
Gunma University, Japan—J Horiguchi, H Takei.
Guy's Hospital, London, UK—I S Fentiman, J L Hayward, R D Rubens, D Skilton.

Heidelberg University I, Germany—H Scheurlen.
Heidelberg University II, Germany—M Kaufmann, H C Sohn.
Helios Klinikum Berlin-Buch, Germany—M Untch.
Hellenic Breast Surgeons Society, Greece—U Dafni, C Markopoulos.
Hellenic Cooperative Oncology Group, Athens, Greece—C Bamia, G Fountzilas, G-A Koliou, K Manousou.
Hellenic Oncology Research Group, Greece—D Mavroudis.
Helsinki Deaconess Medical Centre, Finland—P Klefstrom.
Helsinki University, Finland—C Blomqvist, T Saarto.
Hospital del Mar, Barcelona, Spain—M Gallen.
Humanitas Cancer Center, Milan, Italy—G. Canavese, C Tinterri.
Innsbruck University, Austria—R Margreiter.
Institut Claudius Regaud, Toulouse, France—B de Lafontan, J Mihura, H Roché.
Institut Curie, Paris, France—B Asselain, R J Salmon, J R Vilcoq.
Institut Curie - Hôpital René Huguenin, Paris, St Cloud, France—E Brain, B de La Lande, E Mouret-Fourme.
Institut Gustave-Roussy, Paris, France—F André, R Arriagada, S Delaloge, C Hill, S Koscielny,S Michiels, C Rubino.
Institute of Cancer Research Clinical Trials and Statistics Unit (ICR-CTSU, NCRI), UK—R A'Hern, J Bliss, P Ellis, L Kilburn, J R Yarnold.
Instituto de Investigación Sanitaria Gregorio Marañón, Universidad Complutense, GEICAM, CIBERONC, Madrid, Spain—M Martin.
Integraal Kankercentrum, Amsterdam, Netherlands—J Benraadt, M Kooi, A O van de Velde, J A van Dongen, J B Vermorken.
International Breast Cancer Study Group (IBCSG), Bern, Switzerland—M Castiglione, A Coates, M Colleoni, J Collins, J Forbes, R D Gelber, A Goldhirsch, J Lindtner, K N Price, M M Regan, C M Rudenstam, H J Senn, B Thuerlimann.
International Collaborative Cancer Group, Charing Cross Hospital, London, UK—J M Bliss, C E D Chilvers, R C Coombes, E Hall, M Marty.
International Drug Development Institute, Louvain-la-Neuve, Belgium—M Buyse.
International TABLE Study Group, Berlin, Germany—K Possinger, P Schmid, M Untch, D Wallwiener.
ISD Cancer Clinical Trials Team (incorporating the former Scottish Cancer Therapy Network), Edinburgh, UK—L Foster, W D George, H J Stewart, P Stroner.
Israel NSABC, Tel Aviv, Israel—R Borovik, H Hayat, M J Inbar, T Peretz, E Robinson.
Istituto Nazionale per lo Studio e la Cura dei Tumori, Milan, Italy—T Camerini, F Formelli, G Martelli, M G Di Mauro, P Valagussa.
Istituto Nazionale Tumori IRCCS Fondazione Pascale, Napoli, Italy—F Perrone.
Istituto Scientifico Romagnolo per lo Studio e la Cura dei Tumori, Meldola, Italy—D Amadori.
Italian Cooperative Chemo-Radio-Surgical Group, Bologna, Italy—A Martoni, F Pannuti.
Italian Oncology Group for Clinical Research (GOIRC), Parma, Italy— R Camisa, A Musolino, R Passalacqua.
Japan Clinical Oncology Group–Breast Cancer Study Group, Japan—H Iwata, T Shien.
Japanese Foundation for Multidisciplinary Treatment of Cancer, Tokyo, Japan—O Abe, T Ikeda, K Inokuchi, K Kikuchi, K Sawa.
Kawasaki Medical School, Japan—H Sonoo.
Klinikum Bayreuth, Germany—M Sadoon, A H Tulusan.
Kobe Breast Cancer Oncology Group, Japan—N Kohno, M Miyashita, S Takao.
Korean Cancer Study Group (KCSG), Seoul, South Korea—J.-H Ahn, K H Jung.
Krakow Institute of Oncology, Poland—S Korzeniowski, J Skolyszewski.
Kumamoto University Group, Japan—M Ogawa, J Yamashita.
Leiden University Medical Center, Netherlands—E Bastiaannet, G J Liefers, C J H van de Velde.
Leuven Akademisch Ziekenhuis, Gasthuisberg, Belgium—R Christiaens, P Neven, R Paridaens, W Van den Bogaert.

Ludwig-Maximilians University, Munich, Germany—S Braun.
Marseille Laboratoire de Cancérologie Biologique APM, France—P Martin, S Romain.
Medical University Vienna – General Hospital - Department of Obstetrics and Gynaecology and Department of Medicine I, Vienna, Austria—M Janauer, M Seifert, P Sevelda, C C Zielinski.
Memorial Sloan-Kettering Cancer Center, New York, NY, USA—T Hakes, C A Hudis, L Norton, R Wittes.
Metaxas Memorial Cancer Hospital, Athens, Greece—G Giokas, D Kondylis, B Lissaios.
Mexican National Medical Center, Mexico City, Mexico—R de la Huerta, M G Sainz.
MRC Population Health Research Unit (MRC PHRU), Nuffield Department of Population Health (NDPH), Oxford, UK (ie, members of the MRC PHRU-based EBCTCG Secretariat)—C Boddington, R Bradley, J Braybrooke, J A Burrett, M Clarke, D Cutter, C Davies, D Dodwell, F Duane, V Evans, L Gettins, J Godwin, R Gray, S James, A Kerr, H Liu, E MacKinnon, G Mannu, P McGale, T McHugh, P Morris, H C Pan, R Peto, S Read, C Taylor, Y Wang, Z Wang.
National Cancer Center, Goyang, South Korea—J Ro.
National Cancer Institute, Bethesda, MD, USA—K Camphausen, D Danforth, A Lichter, M Lippman, D Smart, S Steinberg.
National Cancer Institute of Bari, Italy—C D'Amico, M Lioce, A Paradiso.
National Kyushu Cancer Center, Japan—Y Nomura, S Ohno.
National Surgical Adjuvant Breast and Bowel Project (NSABP), Pittsburgh, PA, USA—S Anderson, G Bass, A Brown (deceased), J Bryant (deceased), J Costantino, J Dignam, B Fisher, C Geyer, E P Mamounas, S Paik, C Redmond, S Swain, L Wickerham, N Wolmark.
National Surgical Adjuvant Study Group (N-SAS-BC), Japan—T Aihara, Y Hozumi, Y Nomura.
Nolvadex Adjuvant Trial Organisation, London, UK—M Baum, I M Jackson (deceased), M K Palmer.
North Central Cancer Treatment Group, Mayo Clinic, Rochester, MN, USA—E Perez, J N Ingle, V J Suman.
North Sweden Breast Cancer Group, Umeå, Sweden—A Andresson, N O Bengtsson, H Jonsson, M Sund.
North-West Oncology Group (GONO), Italy—L Del Mastro, M Venturini.
North-Western British Surgeons, Manchester, UK—J P Lythgoe, R Swindell.
Northwick Park Hospital, London, UK—M Kissin.
Norwegian Breast Cancer Group, Oslo, Norway—B Erikstein, E Hannisdal, A B Jacobsen, K V Reinertsen, J E Varhaug.
Norwegian Radium Hospital, Oslo, Norway—B Erikstein, S Gundersen, M Hauer-Jensen, H Høst, A B Jacobsen, R Nissen-Meyer.
Nottingham City Hospital, UK—R W Blamey, A K Mitchell, D A L Morgan, J F R Robertson.
Oita Prefectural Hospital, Japan—H Ueo.
Oncofrance, Paris, France—M Di Palma, G Mathé (deceased), J L Misset.
Ontario Clinical Oncology Group, Hamilton, Canada—M Levine, K I Pritchard, T Whelan.
Osaka City University, Japan—K Morimoto.
Osaka National Hospital, Japan—K Sawa, Y Takatsuka.
Ospedale Policlinico San Martino, Genova, Italy–D Bedognetti, C Bighin, P Bruzzi, L Del Mastro, B Dozin, S Pastorino, P Pronzato, M R Sertoli.
Oxford University Hospitals NHS Foundation Trust, Churchill Hospital, Oxford, UK—E Crossley, A Harris, D Talbot, M Taylor.
Parma Hospital, Italy—G Cocconi, B di Blasio.
Petrov Research Institute of Oncology, St Petersburg, Russia—V Ivanov, R Paltuev, V Semiglazov.
Piedmont Oncology Association, Winston-Salem, NC, USA—J Brockschmidt, M R Cooper.
Pretoria University, South Africa—C I Falkson.
ProBONE study group, Marburg, Germany—P Hadji.
Royal Marsden NHS Trust, London and Sutton, UK—R A'Hern, M Dowsett, A Makris, M Parton, K Pennert, T J Powles, I E Smith, J R Yarnold.
SABRE trial group (international)—G Clack, C Van Poznak.
St George Hospital, Sydney, Australia—L Browne, P Graham.

St George's Hospital, London, UK—J C Gazet.
St Luke's Hospital, Dublin, Ireland—N Corcoran.
Sardinia Oncology Hospital A Businico, Cagliari, Sardinia—N Deshpande, L di Martino.
SASIB International Trialists, Cape Town, South Africa—P Douglas, A Hacking, H Høst, A Lindtner, G Notter.
Saskatchewan Cancer Foundation, Regina, Canada—A J S Bryant, G H Ewing, L A Firth, J L Krushen-Kosloski.
Scandinavian Adjuvant Chemotherapy Study Group, Oslo, Norway—R Nissen-Meyer.
South Sweden Breast Cancer Group, Lund, Sweden—H Anderson, F Killander, P Malmström, L Rydén.
South-East Sweden Breast Cancer Group, Linköping, Sweden—L-G Arnesson, J Carstensen, M Dufmats, H Fohlin, B Nordenskjöld, M Söderberg, M Sundqvist.
South-Eastern Cancer Study Group and Alabama Breast Cancer Project, Birmingham, AL, USA—J T Carpenter.
Southampton Oncology Centre, UK—N Murray, G T Royle, P D Simmonds.
Southwest Oncology Group, San Antonio, TX, USA—K Albain, W Barlow, J Crowley, D Hayes, J Gralow, G Hortobagyi, R Livingston, S Martino, C K Osborne, P M Ravdin.
Stockholm Breast Cancer Study Group, Sweden—J Bergh, T Bondesson, F Celebioglu, K Dahlberg, T Fornander, I Fredriksson, J Frisell, E Göransson, M Iiristo, U Johansson, E Lenner, L Löfgren, P Nikolaidis, L Perbeck, S Rotstein, K Sandelin, L Skoog, G Svane, E af Trampe, C Wadström.
SUCCESS-Study Group, University of Düsseldorf, Germany—W Janni.
Swiss Group for Clinical Cancer Research (SAKK), Bern, and OSAKO, St Gallen, Switzerland—M Castiglione, A Goldhirsch, R Maibach, H J Senn, B Thürlimann.
Tamoxifen Exemestrane Adjuvant Multinational (TEAM) trial—J M S Bartlett, E Bastiaannet, P Hadji, Y Hozumi, D Rea, C J H van de Velde.
Tampere University Hospital, Finland—K Holli, K Rouhento.
Tel Aviv Sourasky Medical Center, Sackler School of Medicine, Tel Aviv University, Israel—T Safra.
Tel Aviv University, Israel—H Brenner, A Hercbergs.
Tokyo Cancer Institute Hospital, Japan—M Yoshimoto.
Toronto-Edmonton Breast Cancer Study Group, Canada—A H G Paterson, K I Pritchard.
Toronto Princess Margaret Hospital, Canada—A Fyles, J W Meakin, T Panzarella, K I Pritchard.
Tunis Institut Salah Azaiz, Tunisia—J Bahi.
UCBG, French Breast Cancer Intergroup UNICANCER, France—S Delaloge, J Lemonnier, A L Martin.
UK Multicentre Cancer Chemotherapy Study Group, London, UK—M Reid, M Spittle.
UK/ANZ DCIS Trial—H Bishop, N J Bundred, J Cuzick, I O Ellis, I S Fentiman, J F Forbes, S Forsyth, W D George, S E Pinder, I Sestak.
UK/Asia Collaborative Breast Cancer Group, London, UK—G P Deutsch, R Gray, D L W Kwong, V R Pai, R Peto, F Senanayake.
University and Istituto Nazionale per la Ricerca sul Cancro, Genoa, Italy on behalf of GROCTA trialists—F Boccardo, A Rubagotti.
University College London, UK—M Baum, S Forsyth, A Hackshaw, J Houghton, J Ledermann, K Monson, JS Tobias.
University Federico II, Naples, Italy—C Carlomagno, M De Laurentiis, S De Placido.
University Medical Center Schleswig-Holstein, Campus Kiel, Germany—C Schem.
University of Edinburgh, UK—L Williams.
University of Leeds, UK—R Bell, D Cameron, R E Coleman, D Dodwell, S Hinsley, H C Marshall.
University of Michigan, USA—D Hayes, L J Pierce.
University of Padua, Padova, Italy—S M M Basso, F Lumachi.
University of Saarland, Germany—E Solomayer.
University of Sheffield, UK—R E Coleman, J M Horsman, J Lester, M C Winter.
University of Texas MD Anderson Cancer Center, Houston, TX, USA—A U Buzdar, L Hsu.
University of Wisconsin, USA—R R Love.

Uppsala-Örebro Breast Cancer Study Group, Sweden—J Ahlgren, H Garmo, L Holmberg, G Liljegren, H Lindman, F Wärnberg.
U.S. Oncology, Houston, USA—L Asmar, S E Jones.
Washington University, St Louis, Missouri, USA—R Aft.
West German Study Group (WSG), Germany—O Gluz, N Harbeck, C Liedtke, U Nitz.
West of Scotland Breast Trial Group, Glasgow, UK—A Litton.
West Sweden Breast Cancer Study Group, Gothenburg, Sweden—A Wallgren, P Karlsson, B K Linderholm.
Western Cancer Study Group, Torrance, CA, USA—R T Chlebowski.
Würzburg University, Germany—H Caffier.
Z-FAST, ZO-FAST & E-ZO-FAST study groups (international)—A M Brufsky, R E Coleman, H A Llombart, on behalf of Novartis Pharmaceuticals.

01 December 2017
